# Design Space Exploration and Machine Learning Prediction of Hydrofluorocarbon Solubility in Ionic Liquids for Refrigerant Separation

**DOI:** 10.1021/acs.jcim.5c01216

**Published:** 2025-08-25

**Authors:** Ashfaq Iftakher, M. M. Faruque Hasan

**Affiliations:** † Artie McFerrin Department of Chemical Engineering, 14736 Texas A&M University, College Station, Texas 77843-3122, United States; ‡ Texas A&M Energy Institute, Texas A&M University, College Station, Texas 77843, United States

## Abstract

Ionic liquids (ILs) are promising solvents for the separation of hydrofluorocarbon (HFC) mixtures due to their tunable solvation properties and negligible vapor pressure. We present a computational study of *R*-32 and *R*-125 solubility in over 341,000 ILs. These HFCs are widely used in refrigerant mixtures such as *R*-410A (50/50 wt % *R*-32 and *R*-125). Using COSMO-RS based molecular simulation, we compute infinite-dilution activity coefficients that reveal a broad spectrum of solubility and selectivity across IL families. Dimensionality reduction techniques, such as PCA and t-SNE, uncover distinct regions in IL design space with varying potential for HFC absorption. While traditional IL selection for *R*-410A separation primarily depends on *R*-32 selective ILs, our analysis reveals many *R*-125 selective ILs. Building on thermodynamic insights, we also propose a new geometric measure for rapid screening of ILs as solvents for *R*-410A separation. Furthermore, we develop machine learning (ML) models that accurately predict infinite dilution activity coefficients of *R*-32 and *R*-125 in ILs. We develop a binary classifier to further distinguish *R*-32- vs *R*-125-selective ILs with over 95% precision and recall. These models provide rapid prediction of infinite dilution activity coefficients, thereby facilitating the identification and design of promising ILs for refrigerant separation involving *R*-32 and *R*-125, and are available at https://github.com/aiftakher/HFC-IL-ActivityCoefficient.

## Graphical abstract

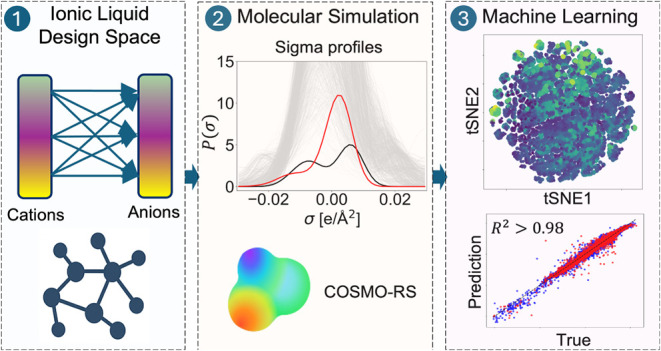

## 1 Introduction

International agreements such as the Kigali Amendment to the Montreal Protocol and the U.S. AIM Act have accelerated global efforts to phase out hydrofluorocarbon (HFC) refrigerants with high global warming potential (GWP) due to their substantial contribution to climate change.^1,2^ As a result, there is a growing demand for technologies capable of reclaiming and recycling existing refrigerants. One of the most prevalent commercial refrigerant mixtures is *R*-410A, which is a near-azeotropic 50:50 wt % blend of difluoromethane (HFC-32 or *R*-32) and pentafluoroethane (HFC-125 or *R*-125). Because of its near-azeotropic nature, *R*-410A cannot be separated effectively using conventional distillation.^3,4^ This challenge has spurred interest in other separation processes including membrane separation^5^ and intensified separation such as extractive distillation using solvents.^6–8^

Ionic liquids (ILs) have emerged as promising candidates as green solvents. Apart from high solubility and selectivity for certain HFCs, ILs also bring desirable solvent properties, including negligible vapor pressure, high thermal stability, and tunable solvation capabilities.^9,10^ Structurally, ILs are composed of bulky organic cations and a diverse set of anions, often functionalized to enhance specific interactions. While the cations and anions have individual positive and negative charges, an IL is neutral and liquid at room temperature. These liquid salt-like molecules often show high solubility for solutes including HFCs.^11^ In particular, several works have demonstrated excellent solubility for *R*-32 and *R*-125 in different ILs.^8,11–15^ At the molecular level, the high solubility of certain HFCs in ILs can be attributed to the distinct polar/nonpolar domains of the ILs as solvents. The nonpolar regions formed by the alkyl side chains interact with the relatively nonpolar HFCs via van der Waals forces. For instance, ILs with fluorinated anions or hydrogen bond donors can enhance solubility for polarizable molecules like *R*-32, while long alkyl chains in the cation may favor *R*-125 solubility.^16,17^

While ILs have shown great promise as solvents, their vast chemical space makes experimental evaluation of each candidate impractical. To that end, computational approaches, particularly those based on the COSMO-RS model, provide an efficient alternative by enabling rapid estimation of key thermodynamic properties such as activity coefficients and Henry’s constants without experimental data. Moreover, leveraging high-throughput simulations allows to identify and explore trends in solubility and selectivity across IL families. While earlier efforts centered on evaluating a few hundred to several thousand ILs,^3,18^ this is a small fraction of the hundreds of thousands of ILs accessible via the combinatorial generation of all possible cation–anion pairs.^19^ Consequently, many potentially high-performing ILs remain unexplored, and the true landscape and extent of IL-selectivity behavior across the chemical space are poorly understood. Moreover, although several ILs are known to preferentially absorb *R*-32,^8,20,21^ the prevalence and structural origins of *R*-125 selectivity are still unclear. Mapping the full spectrum of HFC solubility and selectivity in ILs is therefore essential for both fundamental understanding and to enable high-throughput screening for practical solvent design.^22,23^

Predicting solubility and selectivity of HFCs in ILs is further complicated by the computational cost of reliable quantum-chemical methods such as COSMO-RS, which can require significant resources per IL candidate.^12,16^ While accurate, such methods do not scale well for exhaustive screening of large chemical libraries. To that end, Machine learning (ML) offers a promising alternative, allowing rapid and accurate property prediction once trained on reliable experimental/simulation data.^3,24,25^ Yet, past ML-based studies have been constrained by limited data sets and a narrow focus on common cation–anion pairs. Furthermore, they have rarely addressed inverse selectivity, where an IL favors *R*-125 over *R*-32, a potentially important consideration for process optimization. These limitations highlight the need for a systematic, large-scale study of IL solubility behavior using physically grounded simulation methods (e.g., COSMO-RS) in tandem with ML. By integrating these tools, we can identify new trends, uncover rare but valuable IL candidates, and build models that accelerate future IL discovery without repeated quantum simulations.

In this work, we develop an extensive computational model for HFC solubility in ILs, evaluating over 341,000 ILs and salts generated from 683 unique cations and 505 anions. Using COSMO-RS molecular simulations, we compute infinite-dilution activity coefficients and Henry’s constants for both *R*-32 and *R*-125, enabling us to quantify solubility and selectivity trends across diverse IL families. We uncover distinct structural patterns associated with *R*-125 selectivity, previously underexplored in the literature. To navigate this high-dimensional chemical space, we employ sigma profiles-based molecular descriptors and apply dimensionality reduction techniques (such as PCA and t-SNE) to reveal clustering behavior correlated with solvation properties. Building on these insights, we train ML models that predict infinite dilution activity coefficients and classify IL selectivity with high accuracy. Finally, we propose a geometric screening metric that accounts for solubility gap rather than selectivity alone, providing a potentially more robust criterion for identifying ILs suitable for *R*-410A separation. Based on this metric, we identify a set of potential ILs for *R*-410A separation from each of the major cationic families. Overall, this work demonstrates the systematic use of data science and ML to navigate large molecular design space towards significantly reducing the computational cost of future IL screening for refrigerant separation. The key contributions of this work include:

- A comprehensive COSMO-RS-based screening of over 341,000 ILs and salts for solubility and selectivity toward *R*-32 and *R*-125, identifying structural motifs correlated with high selectivity.
- Application of dimensionality reduction techniques (PCA and t-SNE) to uncover the latent structure of IL design space and visualize solubility/selectivity clusters.
- Development of ML models, including both regression and binary classification, that accurately predict infinite dilution activity coefficients and distinguish between *R*-32- and *R*-125-selective ILs with high precision and recall.
- Proposal of a geometric screening metric based on the angular separation of solubility isotherms to identify ILs that offer significant solubility gaps and improved separation potential over conventional selectivity-based methods.

The rest of this manuscript is organized as follows. In Section 2, we detail the computational methods, including the COSMO-RS framework, dimensionality reduction techniques, and ML model architecture. Section 3 presents the results and discusses the insights into solubility trends, structural clustering of ILs, and performance of the ML models. It also highlights the application of a geometric screening metric to identify high-performing ILs for *R*-410A separation, followed by concluding remarks in Section 4.

## 2 Methods

### 2.1 Data Set Description and Property Calculation

We compute the solubility and selectivity of *R*-32 and *R*-125 for 341,687 ILs and salts, generated by combining 683 cations and 505 anions. For each IL, we compute the infinite-dilution activity coefficients $\gamma_{i}^{\infty }$ of *R*-32 and *R*-125 using the COSMO-RS model at 298.15 K. From the computed infinite dilution activity coefficients and known saturation vapor pressures $P_{i}^{sat}$ of the solutes, we estimate the Henry’s constants as follows:^26^

1 $$H_{i}=\gamma_{i}^{\infty }P_{i}^{sat}$$

The selectivity *S_ij_* of component *i* over *j* is then defined as

2 $$S_{ij}={\left(\frac{H_{j}}{H_{i}}\right)}_{T}$$

where *i* = *R*-32, *j* = *R*-125 (or vice versa). The selectivity quantifies the preferential solubility of one refrigerant over the other in a given IL. A breakdown of the considered data set of ILs across six major cationic families (imidazolium, piperidinium, pyrrolidinium, morpholinium, pyridinium, ammonium), the number of cations in each cationic family, and the maximum observed *R*-32 selectivity are given in Table S2. We notice that the imidazolium family contains the most ILs with the greatest diversity in terms of the number of cations. This family also exhibits the maximum *S_R_*_32_. A miscellaneous family contains the most of number of ILs and salts and demonstrates extremely high *R*-32 selectivity.

### 2.2 Quantum Chemical Calculations with COSMO-RS

The conductor-like screening model for real solvents (COSMO-RS)^27–29^ combines quantum chemistry and thermodynamic principles to estimate thermophysical properties such as liquid-phase chemical potentials, activity coefficients and solubility without requiring binary experimental data or fitted parameters. COSMO-RS first performs a quantum chemical calculation on each molecule in a virtual conductor to determine the sigma surface charge density distribution, also known as the σ-profile. This σ-profile, denoted *p*(σ), represents the probability distribution of screening charge densities (in e/Å^2^) over the surface of a molecule. The residual chemical potential $\mu_{i}^{S}$ of solute *i* in solvent *S* is calculated by integrating the interaction of *p_i_* (σ) with the screening charge environment of the solvent μ*^S^* (σ):

3 $$\mu_{i}^{S}=\int p_{i}(\sigma )\mu^{S}(\sigma )d\sigma$$

The infinite dilution activity coefficient $\gamma_{i}^{\infty }$ is then derived from the residual chemical potential $\mu_{i}^{S}$ using

4 $$\ln\gamma_{i}^{\infty }=\frac{\mu_{i}^{S}}{RT}$$

where *R* is the universal gas constant and *T* is the temperature. Each IL has one σ-profile for cation and another σ-profile for anion, with 50 charge-density bins. These individual profiles are first obtained for the cations and anions at the BP/TZVP-FINE level in COSMOtherm 2023 with default parameters and are then summed point-wise to obtain a 50-dimensional descriptor vector representing the IL. We use all 50 bins as input features, each of which corresponds to a physically meaningful surface-charge interval. Using the σ-profiles, the infinite dilution activity coefficients of *R*-32 and *R*-125 are computed.

### 2.3 Dimensionality Reduction of IL Design Space

The sigma profile of each IL is constructed by pointwise summation of the 50-bin σ-profiles of its cation and anion. This high-dimensional representation encodes molecular polarity information and is used to explore latent structure in IL chemical space. To uncover latent structure and visualize clustering based on solubility behavior, we apply Principal Component Analysis (PCA)^30^ and t-distributed Stochastic Neighbor Embedding (t-SNE).^31^ Let $X\in R^{n\times d}$ be the matrix of *n* ILs with *d* = 50 sigma-profile features. PCA projects ***X*** to a lower-dimensional space via eigendecomposition of the covariance matrix:

5 $$Cov\left(X\right)=\frac{1}{n}X^{T}X$$

The top eigenvectors form a basis $W_{k}\in R^{d\times k}$ which contain *k* leading eigenvectors and used for projection as follows:

6 $$Z_{PCA}=X\cdot W_{k}$$

We chose *k* =2 to enable 2D visualization of IL clusters by solubility and selectivity. To capture nonlinear relationships, we employ t-SNE, which minimizes the Kullback–Leibler (KL) divergence between high-dimensional and low-dimensional pairwise similarities. For a pair of high-dimensional data points *x_i_*,*x_j_*, the conditional probability is:

7 $$p_{j|i}=\frac{\exp\left(-\frac{{||x_{i}-x_{j}||}^{2}}{2\sigma_{i}^{2}}\right)}{\underset{k\neq i}{\sum }\exp\left(-\frac{{||x_{i}-x_{k}||}^{2}}{2\sigma_{i}^{2}}\right)}$$

t-SNE seeks a low dimensional mapping *y_i_* such that the following KL divergence is minimized:

8 $$KL\left(P||Q\right)=\underset{i}{\sum }\underset{j}{\sum }p_{ij}\log\left(\frac{p_{ij}}{q_{ij}}\right)$$

where *q_ij_* are the low-dimensional pairwise similarities (in the embedding space) that are modeled via a Student’s t-distribution.

In this work, both PCA and t-SNE are implemented using scikit-learn Python library with standard settings. These dimensionality reduction techniques enable to identify structural motifs correlated with HFC selectivity, such as the emergence of *R*-125 selective clusters in morpholinium and pyrrolidinium families (described later).

### 2.4 Machine Learning Models

We develop regression and classification models using feedforward artificial neural networks (ANNs) trained on sigma profiles as molecular descriptors. Each input feature of an IL corresponds to a 50-dimensional vector $x\in R^{50}$ , constructed by summing the cation and anion sigma profiles.

#### 2.4.1 Regression: Infinite Dilution Activity Coefficient Prediction

The regression model predicts the infinite dilution activity coefficient ŷ∈R for *R*-32 and *R*-125 from sigma profiles:

9 ŷ=f(x;θ)

where ***f*** is a neural network with two hidden layers (64 neurons each), ReLU activation, and linear output. The network has trainable weight and bias parameters ***θ*** which are trained by minimizing mean squared error (MSE):

10 $$L_{MSE}=\frac{1}{N}\overset{N}{\underset{i=1}{\sum }}{\left({ŷ}_{i}-y_{i}\right)}^{2}$$

We use the Adam optimizer^32^ with early stopping based on validation error.

#### 2.4.2 Classification: *R*-32 Selective vs *R*-125 Selectivity

To classify ILs as *R*-32 selective vs *R*-125 selective, we train a binary classifier using the same architecture for regression bbut with sigmoid activation at the output:

11 $$ŷ=\sigma \left(W_{2}\cdot ReLU\left(W_{1}\cdot x+b_{1}\right)+b_{2}\right)$$

We minimize the binary cross-entropy loss:^33^

$$L_{BCE}=-\frac{1}{N}\overset{N}{\underset{i=1}{\sum }}\left[y_{i}\;\log{ŷ}_{i}+\left(1-y_{i}\right)\log\left(1-{ŷ}_{i}\right)\right]$$

## 3 Results and Discussion

### 3.1 Solubility and Selectivity of HFCs in ILs

We use COSMO-RS,^27,28^ a quantum-chemistry-based thermodynamic model, to predict *R*-32 and *R*-125 solubility in ILs. COSMO-RS has been established as a reliable *a priori* method for predicting activity coefficients and phase equilibria without fitted binary parameters.^12,16^ A key advantage of COSMO-RS is its ability to screen arbitrary cation–anion combinations, capturing quantum-mechanical effects and hydrogen-bonding, unlike group-contribution models^34,35^ that require extensive parametrization. To justify the use of COSMO-RS, we compare predicted Henry’s constants against experimental values for ∼30 ILs (Table  S1 and Figures S1,S2). The resulting Pearson R^2^ values of 0.75 for *R*-32 and 0.88 for *R*-125 indicate that COSMO-RS captures qualitative solubility trends well at infinite dilution, which is consistent with previous studies.^3,4,16,18^ Also note that we pick 298.15 K as the temperature for evaluating solubility, as most solvents in physical separation processes are fed at near this condition.^6,22,36^ Additionally, this allows us to benchmark with other studies that also use 298.15 K as the reference temperature for *R*-410A applications.^3,18^

In this work, we compute infinite-dilution activity coefficients $\left(\gamma_{i}^{\infty }\right)$ , Henry’s constants $\left(H_{i}\right)$ and Selectivity (*S_ij_*) for *R*-32 and *R*-125 at 298.15 K across a data set of 341,687 ILs and salts generated from 683 cations and 505 anions (see Methods section for details). The superset of cations includes all major IL families (imidazolium, piperidinium, pyrrolidinium, morpholinium, pyridinium, and ammonium) with diverse functional groups (Table S2). Anions range from simple inorganic anions to functionalized organic anions (e.g., halides, sulfonates, carboxylates, etc.). Imidazolium-based ILs, one of the largest cationic families, yield many highly *R*-32-selective ILs (*S_R_*_32_ > 30). The is driven by a combination of electrostatic interactions and hydrogen bonding with the C–H bond of *R*-32. Pyridinium ILs also contribute a significant number of *R*-32-selective ILs, while ammonium-based ILs with long alkyl chains favor *R*-125 absorption.

Figure 1 shows the distribution of Henry’s constants for *R*-32 and *R*-125 across 341,687 ILs and salts. ILs with the highest *R*-32 absorption have *H_R_*_32_ between ∼1 to 5 MPa, while some ILs exhibit poor solubility for either *R*-32 or *R*-125 (*H*_R32_,*H_R_*_125_ > 100 MPa). The inset highlights ILs with both high *R*-32 solubility (*H_R32_* ≤ 10 MPa) and notable selectivity. At low Henry’s constants (<2 MPa), *S_R_*_32_ does not exceed 10, indicating a trade-off between *R*-32 solubility and selectivity. Most ILs are *R*-32 selective (*S_R_*_32_ > 1), likely due to *stronger interactions between ILs and R-32 (two C–H bonds) than with R-125 (one C–H bond and more heavily fluorinated structure)*. Still, ILs with *S_R_*_32_ < 1 exist, demonstrating ILs with *R*-125 selectivity is achievable with suitable structure.

**Figure 1:**
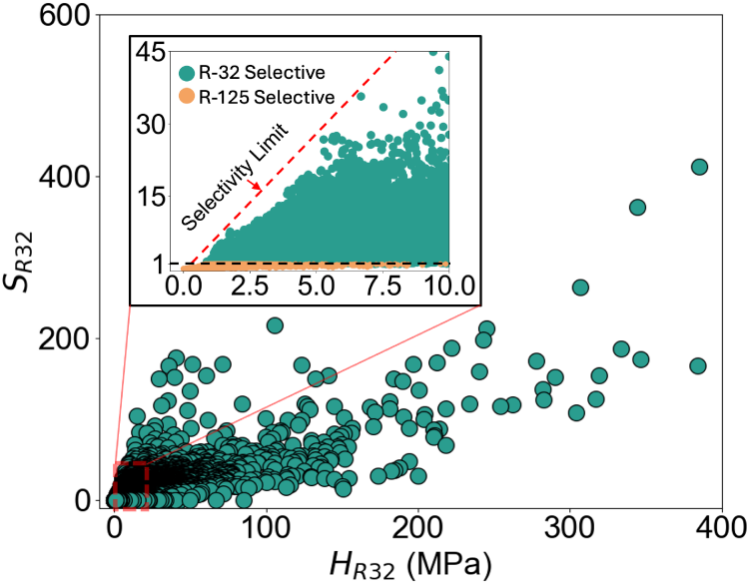
COSMO-RS predicted Henry’s constant and selectivity of *R*-32 in ILs. The inset shows ILs with high *R*-32 absorption (*H_R32_* ≤ 10 MPa). *R*-125 selective ILs have *SR32* < 1. A thermodynamic limit exists on the maximum *R*-32 selectivity among ILs with high *R*-32 absorption.

### 3.2 Structural Insights via Dimensionality Reduction

To understand how IL structure influences solubility behavior, we analyze sigma (σ)-profiles of ILs and apply unsupervised dimensionality reduction. As shown in Figure 2A (two vertical dashed lines at σ = ± 0.0084 e/Å^2^), σ of *R*-32 and *R*-125 separates surface charge into negative polar, nonpolar and electron accepting regions, respectively.^16,37^ We apply PCA to the sigma profiles of all ILs (Figure 2B), where the first two components (PC1 and PC2) capture ∼30% of the variance in the original 50-dimensional sigma profiles space. Clusters observed in PCA correlate with IL selectivity. To uncover nonlinear patterns and more distinct subclusters, we also apply t-SNE (Figure 3), which projects the high-dimensional sigma profiles into two latent dimensions (t-SNE1 and t-SNE2), preserving local distance relationships. Clusters with tight groups of points indicate ILs with similar σ-profiles, while distant points reflect structural differences. Notably, all cationic families exhibit regions with high *R*-32 selective ILs. Piperdinium, Pyrrolidinium and Morpholinium exhibit greater structural diversity. For *R*-125 selective ILs (Figures S7 and S8), clustering is more distinct across cationic families. Morpholinium and Pyrrolidinium ILs form localized high selectivity zones, pointing to isolated ILs with extremely high *R*-125 selectivity.

**Figure 2:**
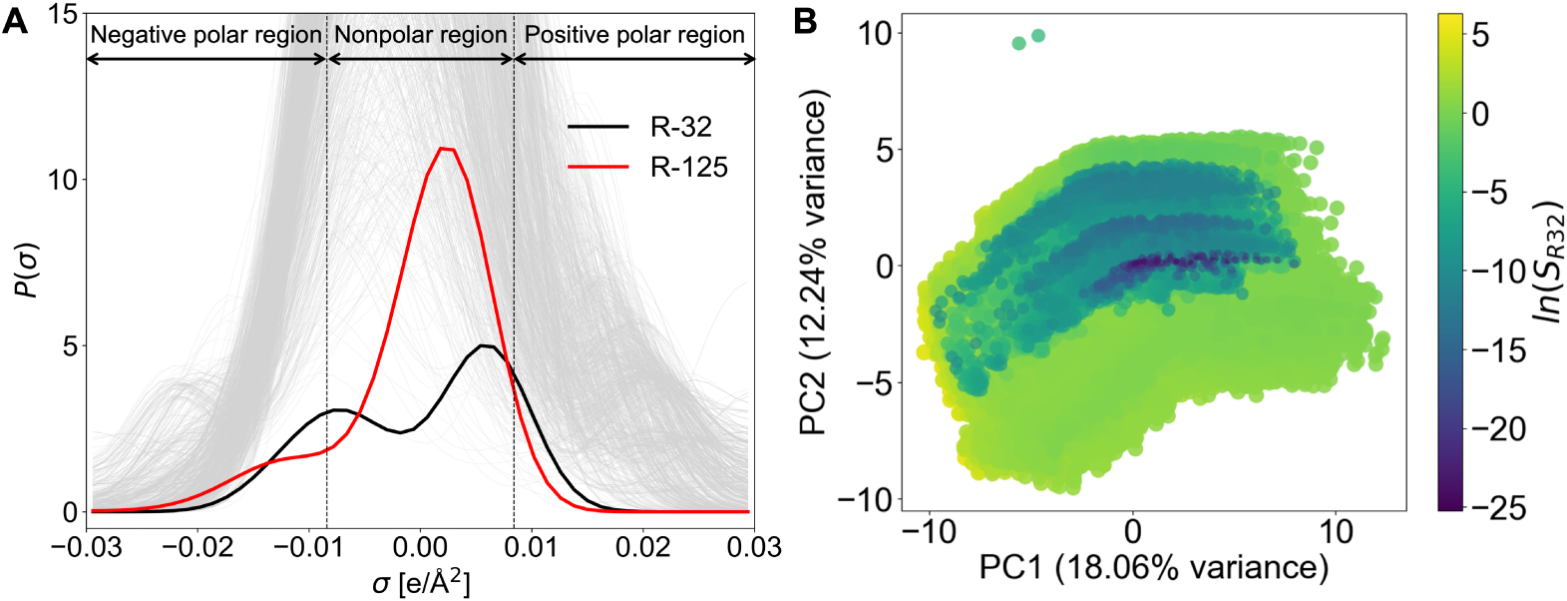
(A) Sigma profiles of *R*-32 and *R*-125 with 1000 randomly selected ILs (shown in light gray). (B) A two-dimensional projection of learned embeddings using PCA. Each point denotes an IL and is colored by the natural log of *R*-32 selectivity.

**Figure 3:**
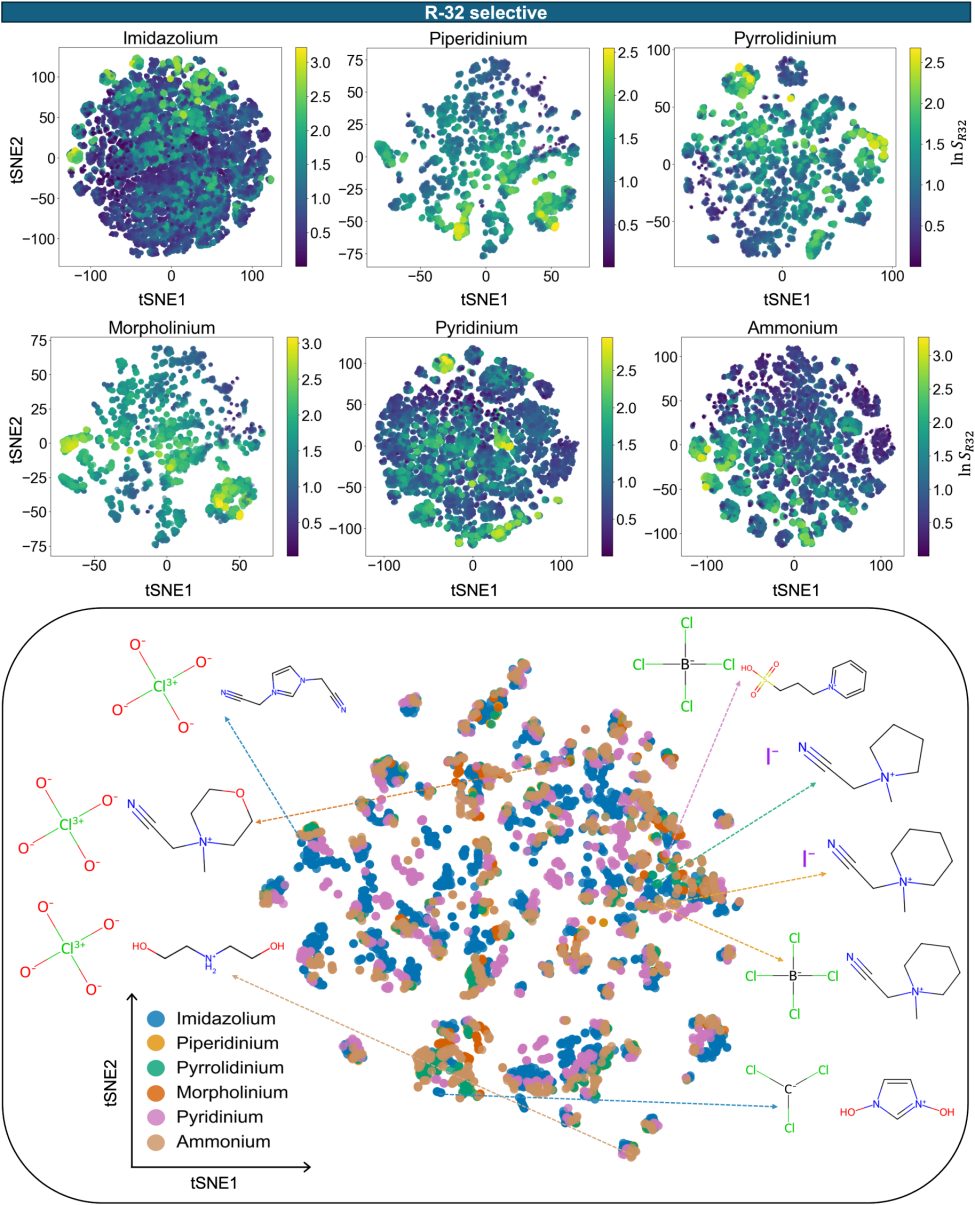
A two-dimensional projection of sigma profiles-based embeddings of IL chemical space using t-SNE. Each class of cationic family is colored by *R*-32 selectivity values. The structures of top ILs from each cationic family are annotated with their structures in the embedding space. For visualization, top 5000 *R*-32 selective ILs are chosen with, *H*_*R*32_ ≤ 10 MPa and *S*_*R*32_ > 1.

### 3.3 Predictive Modeling of Solubility Using ML

Given the strong relationship between sigma profiles and gas solubility, we develop ML models to predict infinite dilution activity coefficients. Sigma profiles, which encode molecular polarity information, serve as effective features for ML^38^ and enable rapid screening of IL candidates. Once trained, these models can bypass costly COSMO-RS calculations. By integrating COSMO-RS with data-driven modeling, we seek to provide both physical insight (via thermodynamic analysis) and predictive tools via ML. We use a feedforward ANN trained on sigma profiles of 341,687 ILs to predict ln γ^∞^ for *R*-32 and *R*-125. The input to the network is the concatenated sigma profile (with 50 intervals) of the IL (see details in Section 2). The model architecture involves two hidden layers with 64 nodes each. We employ rectified linear unit (ReLU) as the activation function for all nodes in the hidden layers and a linear function in the output layer. The model is trained with Adam optimizer^32^ that employs stochastic gradient descent to minimize the mean squared error (MSE). We set a learning rate of 1 × 10^– 3^, batch size of 32 and other default settings in TensorFlow. The data set involving 341,687 ILs is split into 64% for training, 16% for validation and 20% for testing. Data set partitioning details and summary statistics for the training, validation, and test sets for all three regression models are provided in Table S4. As shown, the means and standard deviations (SD)­of each target are effectively identical across splits, confirming no distributional shift. In addition to the 64/16/20 hold-out split, we perform 5-fold cross-validation on the 80% (training+validation) data for the regression model for selectivity. The fold-wise metrics (mean ± SD) are given in Table S3. For example, the mean R^2^ value is found to be 0.984 ± 0.001, and the RMSE is 0.204 ± 0.009. The small SD in both R^2^ and RMSE values demonstrate stable performance of the regression models obtained for selectivity.

Figure 4 shows parity plots comparing predicted and COSMO-RS-calculated infinite dilution activity coefficients, ln γ^∞^. For both *R*-32 (Figure 4A) and *R*-125 (Figure 4B), most data points lie near the diagonal, indicating excellent agreement. The *R* ^2^ on the test set is 0.972 for *R*-32 and 0.983 for *R*-125, with very low root-mean-square error. This level of accuracy translates to predicted Henry’s constants deviating by only a few percent from COSMO-RS results for most ILs and salts. The model achieves high accuracy across the full range of infinite dilution activity coefficients. For *R*-32 (Figure 4A), it reliably predicts both moderate values (ln γ^∞^ ≈ 0) and large values (ln γ^∞^ > 5), with minimal systematic bias. Only at the extreme tail where COSMO-RS predicts extraordinarily large γ^∞^ values, slightly increased prediction error is observed. Parity plots have slopes near unity, and intercepts close to zero, further confirming that the ReLU-ANN model is neither overpredicting nor under-predicting. Figure 4C shows the prediction of selectivity on a logarithmic scale. Balanced visualization of *S_R_*_32_ > 1 vs *S*_R32_ < 1 is possible on a log scale. On a linear axis, all *R*-125–selective ILs (*S_R_*_32_ < 1) collapse into [0, 1], thereby making it impossible to distinguish those with very small *R*-32 selectivity (e.g., *S_R_*_32_ ≈ 0.01). On the other hand, a log scale (ln *S_R_*_32_ >0 for *R*-32 selective, ln *S_R_*_32_ < 0 for *R*-125 selective) displays types of selectivity and also highlights outliers in each class. Detailed statistical indicators of the log-selectivity model (Figure 4C) are given in Section S5. When trained on all ILs without PF^_6_^ anion, the ANN model for *R*-32 selectivity predicts PF^_6_^-based IL selectivities with R^2^ = 0.826 and RMSE = 0.255 (Section S5 and Figure s S12,S13), demonstrating strong extrapolative capability to an entirely new anion family. To identify the parts of the σ-profile that mostly influence the ANN predictions, we employ approximate SHapley Additive exPlanations (SHAP^39^) values on standardized inputs (bins p1–p50). We use a background sample of 512 training points and an evaluation sample of 1,000 test points. Global importance is then summarized as the mean absolute SHAP value per bin. SHAP analysis indicates that predictions are driven by a compact subset of σ-bins. The top-10 and top-20 bins account for 40.2% and 68.9% of total attribution, respectively. The effective number of features is 38.4 (entropy), which is below the full 50 bins. The top-10 leading bins are 45th, 37th, 22nd, 30th, 33rd, 40th, 46th, 42nd, 31st, and 35th. Details and figures are provided in the Supporting Information (Section S5, Figures S15–S17 and Table S7).

**Figure 4:**
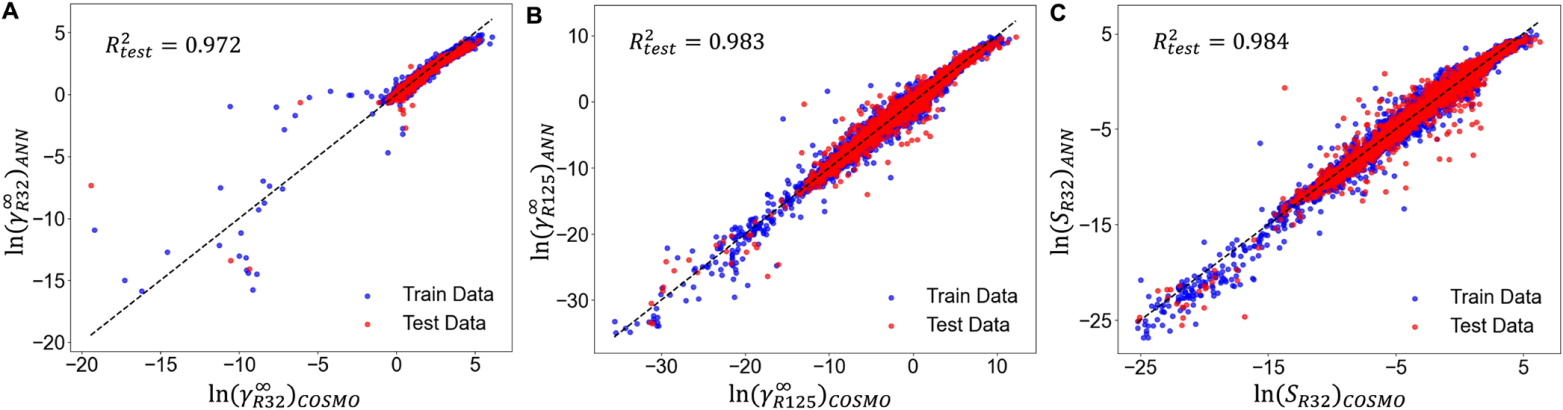
Performance of ML models in predicting the infinite dilution activity coefficients of *R*-32 and *R*-125 in ILs. ReLU-ANN models exhibit highly accurate prediction of infinite dilution activity coefficients of *R*-32 and *R*-125 in ILs. A similar ReLU-ANN model also achieves highly accurate prediction of *R*-32 and *R*-125 selectivity of ILs.

This strong performance stems from the sigma profiles, which capture essential intermolecular interaction. The neural network learns nonlinear correlations and produces a model that, in milliseconds can predict the solubility (Henry’s constant) of both *R*-32 and *R*-125 in ILs with an accuracy comparable to running a full quantum-chemical COSMO-RS calculation .

In addition to predicting infinite dilution activity coefficients, we develop a binary classification model to predict IL selectivity type (*R*-32 selective vs *R*-125 selective). We define ILs with *S_R_*_32_ ≥ 1 as *R*-32 selective; otherwise, they are *R*-125 selective. The ANN architecture of the binary classification model remains the same as the ReLU-ANN, but the output activation function is now changed to sigmoid, making it suitable for binary classification. We employ binary cross entropy as the loss function. The model achieves training and testing accuracies of 0.996 and 0.995, respectively. We interpret the classification model in terms of precision (*P*), recall (*r*), F1-score (*F*1), and support. We denote precision, true positive, false positive, and false negative as *P*, *TP*, *FP*, *FN* respectively. Then, $P=\frac{TP}{TP+FP}$ , $r=\frac{TP}{TP+FN}$ , and $F1=2\cdot \left(\frac{P\cdot r}{P+r}\right)$ . Support is the number of actual occurrences of the class in the data set. Precision is the ratio of true positives to the sum of true positives and false positives. It measures the accuracy of positive predictions. Recall is the ratio of true positives to the sum of true positives and false negatives. It measures the ability of the model to find all positive instances. F1-Score is the harmonic mean of precision and recall. It balances the two metrics.

Table 1 presents the classification report of this model, including the precision, recall, and F1-score for each class, as well as the overall accuracy. For *R*-32-selective ILs, the precision is ∼1 and recall are ∼ 1, meaning that the classifier achieves near perfect labels and finds nearly all true *R*-32-selective ILs. The F1-score for the *R*-32 class is also near unity, reflecting the strong performance. For the *R*-125-selective class, precision and recall are 0.95 and 0.95, respectively. The slightly lower recall for *R*-125 class suggests the model might misclassify a small number of *R*-125-selective ILs as *R*-32-selective. This may be because those ILs have sigma profiles that are borderline similar to some ILs that are *R*-32 selective. Class-balance details for the binary classifier (*R*-32 vs *R*-125 selective) in each data set split are provided in Table S5. As shown in Table S5, class proportions are preserved in Train (64%), Validation (16%), and Test (20%), and no additional sampling (over- or under-sampling) or class-weighting is applied. We monitor overfitting by comparing training and validation loss curves (Figure S14). We also perform 5-fold cross-validation and train a regularized variant with Dropout (*p* = 0.2) and L2 penalty (λ = 1 × 10^–4^). As shown in Figure S14, the training and validation losses track closely, with no sign of divergence. On the held-out test set, the baseline classifier achieves an accuracy of 0.995, a precision of  0.997, a recall of 0.998, and an AUC of 0.999. Incorporating dropout and L2 regularization yields comparable results (Accuracy = 0.992; Precision = 0.994; Recall = 0.997; AUC = 0.999), indicating robust generalization. A 5-fold cross-validation further confirms stability (see Table S6).

**Table 1:** Classification Report for *R*-125 and *R*-32 Selectivity

Class	Precision	Recall	F1-Score	Support
*R*-125 Selective	0.95	0.95	0.95	4062
*R*-32 Selective	1.00	1.00	1.00	64,276

Figure 5 shows the confusion matrix of the classifier model. The results on the test set shows that the diagonal elements (true positives for each class) are significantly larger than off diagonal elements. In the *R*-32-selective ILs, only a small fraction of ILs is misclassified. For *R*-125-selective ILs, 95% of predictions are accurate, despite this class making up only 5.95% of the data. Most misclassifications occur near *S_R_*_32_ ≈ 1, where small errors can flip the predicted class. Despite this, high precision for the *R*-125 class ensures that predictions in this category are highly reliable.

**Figure 5:**
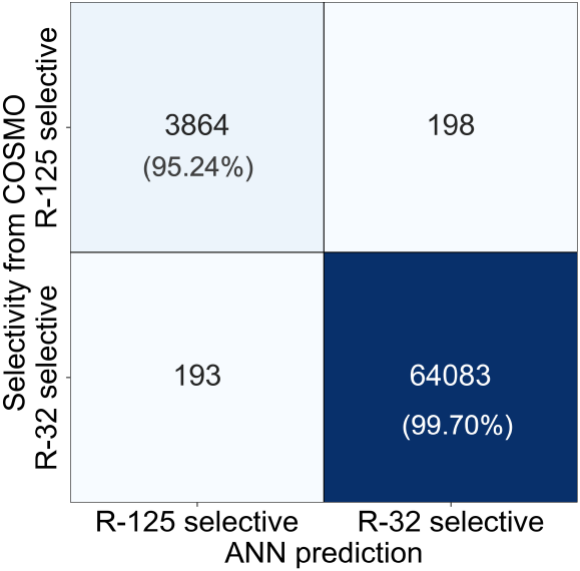
Performance of the ML-based classification model for predicting selectivity.

The classifier effectively learns a nonlinear decision boundary in sigma profile space, separating *R*-32- and *R*-125-selectivity in ILs. In practice, this classifier can serve as a fast-screening tool. The excellent performance implies that the boundary between those regions is well-behaved, and that the sigma profiles help provide a clear boundary. For any new IL, it can instantly predict the selectivity class, bypassing the need for full COSMO-RS calculations. This enables high-throughput filtering, narrowing the search space based on desired separation targets. The method can be extended to multiclass classification (e.g., *R*-32 vs *R*-125 vs *R*-134a) for more complex refrigerant mixtures. To enable rapid estimation of solubility and selectivity of R-32 and R-125 in ILs, we provide the ML models and trained weights on Github: https://github.com/aiftakher/HFC-IL-ActivityCoefficient.

### 3.4 Geometric Perspective on Solubility Gaps

The suitable IL selection for *R*-410A separation poses a multiscale challenge involving both solubility (Henry’s constant) and selectivity (ratio of Henry’s constants).^3,22,40^ Traditional screening based on selectivity alone may lead to suboptimal or infeasible choices.^3^ To illustrate this, consider top 100 ILs and salts selected based on their *R*-32 selectivity (Figure 6). Within this subset, the minimum and maximum *R*-32 selectivity (*S_R_*_32_) are 71.93 and 551.84, respectively. Correspondingly, the Henry’s constants for *R*-32 (*H_R_*_32_) range from a moderate value of 14.66 MPa to an extremely high value of 737.01 MPa (see Figure S9). We further select top five ILs from each major cationic family, with an additional restriction of *H_R32_* ≤ 10 MPa. As shown in Figure S8, this refined set of ILs exhibit a more moderate *R*-32 selectivity values (*S_R_*_32_ ranging from 11.72 to 26.41) and notably lower Henry’s constants (*H_R_*_32_ between 3.96 and 9.64 MPa). Interestingly, our analysis reveal a consistent structural feature among the piperidinium, pyrrolidinium, and morpholinium families, i.e., the presence of a cyanomethyl functional group in the cationic structure. Specifically, 1-(cyanomethyl)-1-methylpiperidinium, 1-(cyanomethyl)-1-methylpyrrolidinium, and 4-(cyanomethyl)-4-methylmorpholinium are present. Importantly, ILs containing the 1-(cyanomethyl)-1-methylpiperidinium cation in combination with tetrachloroborate (BCl4^–^) and tetrachloroferrate­(III)-hexachloride anions exhibited the lowest Henry’s constants for *R*-32.

**Figure 6:**
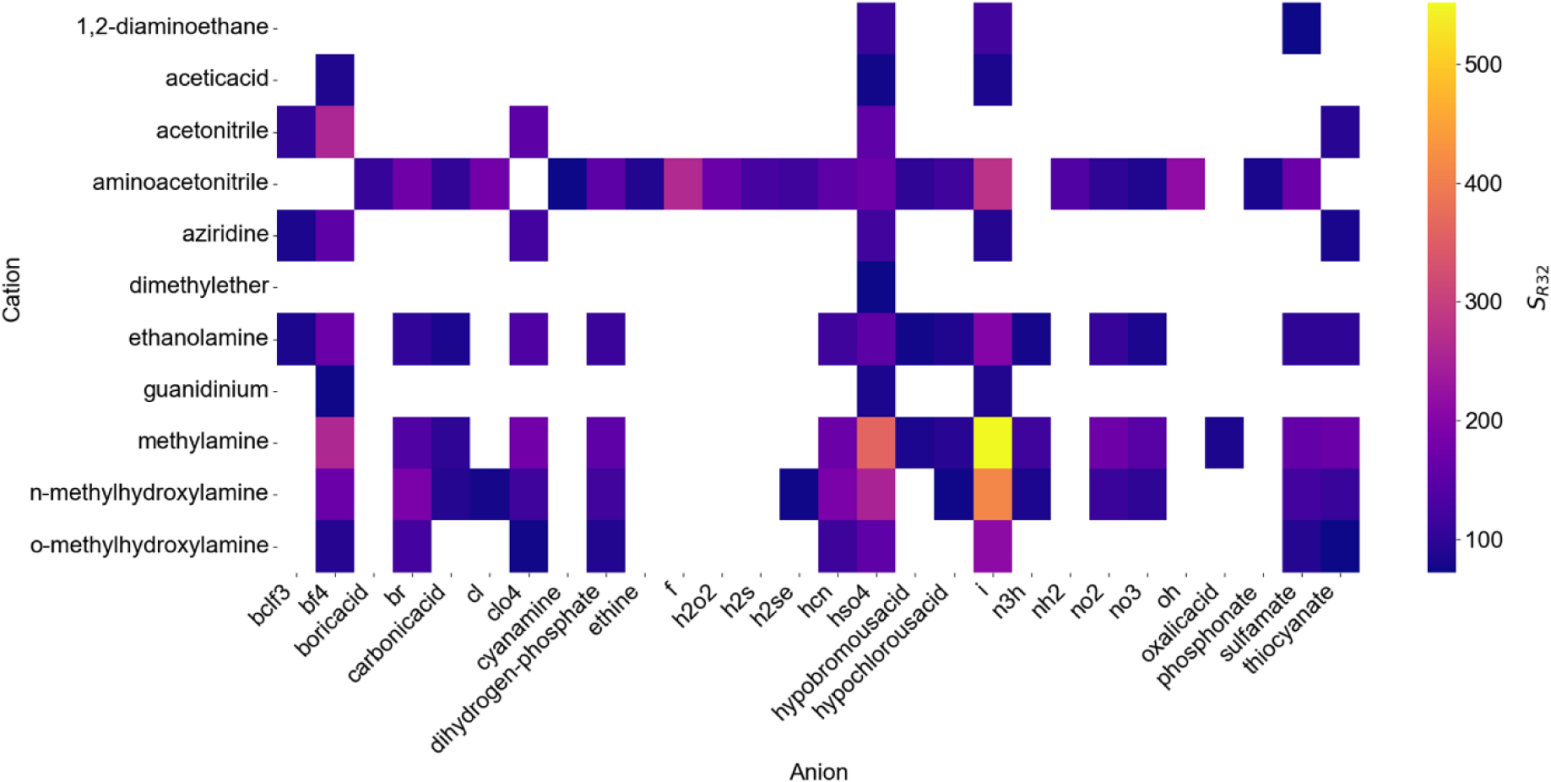
Heatmap of the selectivity of top 100 ILs based on *R*-32 selectivity.

Next, we provide an alternative IL screening metric. For that, consider two solvents for separating *R*-410A (Figure 7A). Solvent 1 (1,2-diaminoethane sulfamate) has high selectivity (*S_R_*_32_ = 71.93) but poor solubility (*H_R_*_32_ = 101.37 MPa, *H_R_*_125_ = 5946.53 MPa). Solvent 2 (1-(2-hydroxyethyl)-3-methylimidazolium trifluoromethyltrifluoroborate) has lower *R*-32 selectivity (*S_R_*_32_ = 5.96) but much better *R*-32 solubility (*H_R_*_32_ = 3.27MPa, *H_R_*_125_ = 19.49MPa). Despite a 12 times lower selectivity, COSMO-RS generated solubility curves (Figure 7A) reveal that solvent 2 offers superior separation. An alternative perspective is to analyze this by computing the angle formed by the solubility curves (near Henry's zone) of *R*-32 and *R*-125 in both solvents with respect to the *x*-axis. In Solvent 1, the *R*-32 and *R*-125 solubility curves are nearly parallel (tan^–1^(101.37) = 89.43°, tan^–1^(5946.53)= 89.99°), indicating poor separation. In Solvent 2, the curves yield a 14.07° difference (tan^–1^(3.27) = 72.99°, tan^–1^(19.49) = 87.06°), indicating more effective separation.

**Figure 7:**
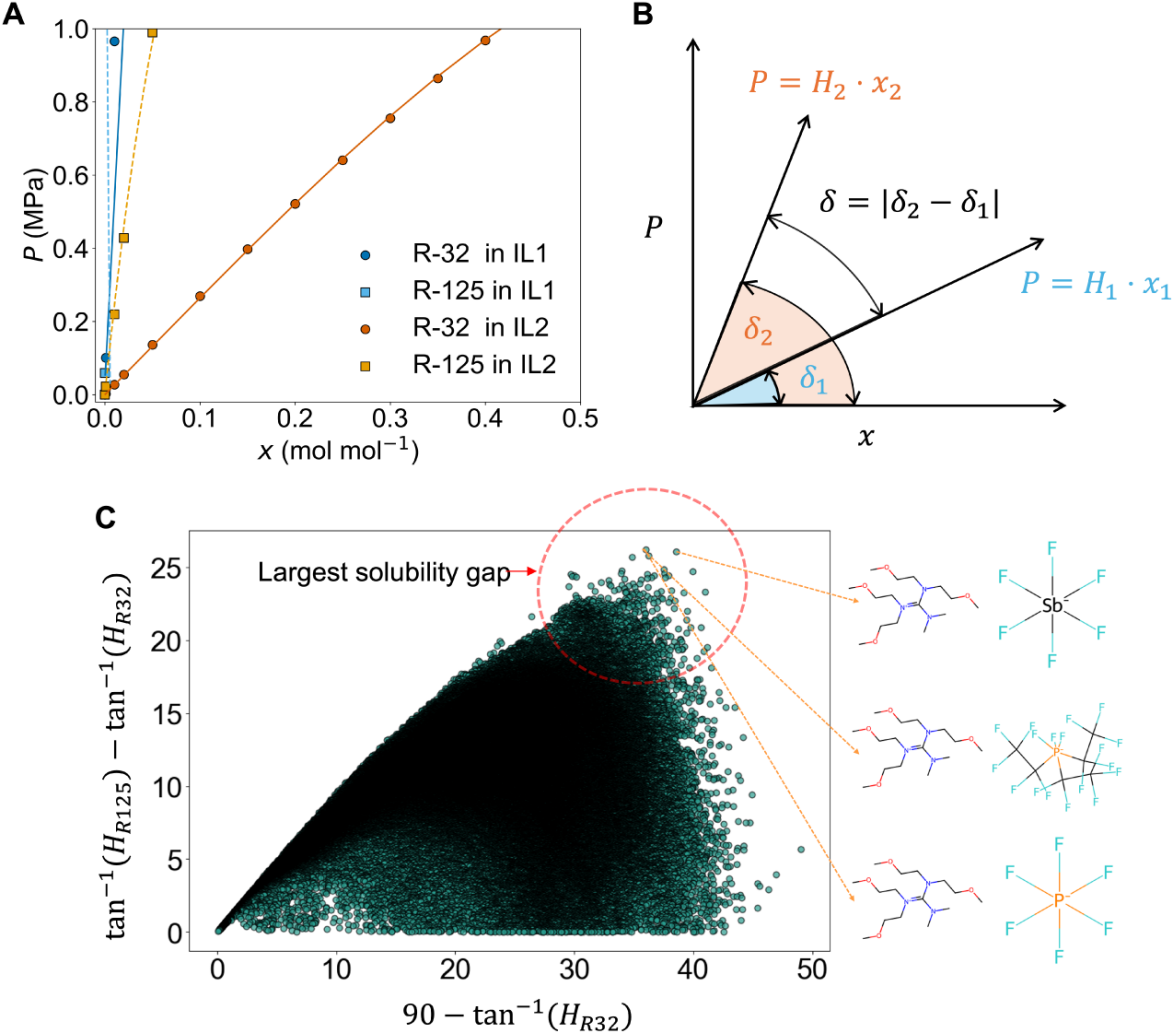
IL selection for *R*-410A separation. (A) *R*-410A solubility in two different solvents at 298.15 K. Here, the liquid–liquid equilibria (LLE) data points are estimated from COSMO-RS. (B) Quantification of the driving force, δ, that takes into account the angles formed by the isotherms of *R*-32 and *R*-125 in ILs. (C) Quantification of the driving force of the entire IL design space reveals sets of ILs with superior solubility gap.

To capture absolute solubility differences, we define a geometric measure, δ, based on the angle between solubility curves (near Henry's zone) derived from Henry’s constants as follows. In an isothermal P-x diagram (Figure 7B), the solubility curve of each solute (*P* =*H* ·*x*) forms an angle with the *x*-axis: δ = tan^–1^(*H*_1_), and δ = tan^–1^(*H*_2_). We quantify the ability of a solvent to distinguish between solutes as follows:

12 $$\delta =\left|\delta_{2}-\delta_{1}\right|$$

where, δ can be interpreted as a driving force for selective absorption of a solute over the other in a mixture. Larger δ implies better separation due to greater differences in mole fractions at fixed pressure. Figure 7C shows this across the IL chemical space using δ. The *x*-axis denotes the values of 90°–tan^–1^(*H_R_*_32_) where higher values indicate a better *R*-32 absorption. The *y*-axis denotes the values of tan^–1^(*H_R_*_125_)–tan^–1^(*H_R_*_32_) where higher values indicate a worse *R*-125 absorption. Therefore, the top-right region represents ILs with both strong *R*-32 absorption and weak *R*-125 absorption, which is ideal for *R*-410A separation. The chemical structures of the top three ILs share a unique cation, di­[n-di­(2-methoxyethyl)]]-n-dimethyl-guanidinium, which has not yet been explored experimentally for *R*-410A separation, highlighting promising directions for discovering new ILs for refrigerant separation. We also identify top five ILs from each of the cationic family based on the newly defined metric, δ which are reported in Table 2.

**Table 2:** Top ILs from Each Cationic Family Based on the Largest δ

Cation	Anion	$H_{R32}$ (MPa)	$H_{R125}$ (MPa)	$$S_{R32}$$	δ	MW (g/mol)
1,3-bis(3-cyanopropyl)imidazolium	diperfluorophenyl-(2,3,5,6-tetrafluorophenyl)-methane	1.42	4.32	3.72	22.08	698.50
1,3-bis(3-cyanopropyl)imidazolium	diperfluorophenyl-(4-methyl-tetrafluorophenyl)-methane	1.42	4.30	3.70	21.97	712.50
1,3-bis(3-cyanopropyl)imidazolium	diperfluorophenyl-(triafluoro-3,5-dichlorophenyl)-methane	1.46	4.51	3.79	21.92	749.40
1-[2-(2-methoxyethoxy)ethyl]-3-methyl-imidazolium	tris(pentafluoroethyl)trifluorophosphate	1.38	3.98	3.55	21.89	630.20
1,3-bis(3-cyanopropyl)imidazolium	triperfluorophenyl-methane	1.38	3.97	3.53	21.81	716.50
1-(3-methoxypropyl)-1-methylpiperidinium	tris(pentafluoroethyl)trifluorophosphate	1.45	4.83	4.10	22.98	617.30
1-(3-methoxypropyl)-1-methylpiperidinium	diperfluorophenyl-(4-methyl-tetrafluorophenyl)-methane	1.41	4.50	3.92	22.82	681.50
1-(3-methoxypropyl)-1-methylpiperidinium	diperfluorophenyl-(triafluoro-3,5-dichlorophenyl)-methane	1.46	4.85	4.07	22.76	718.40
1-(3-methoxypropyl)-1-methylpiperidinium	diperfluorophenyl-(2,3,5,6-tetrafluorophenyl)-methane	1.41	4.43	3.86	22.71	667.50
1-(3-methoxypropyl)-1-methylpiperidinium	triperfluorophenyl-methane	1.38	4.26	3.78	22.69	685.50
1-(3-methoxypropyl)-1-methylpyrrolidinium	diperfluorophenyl-(4-methyl-tetrafluorophenyl)-methane	1.42	4.75	4.11	23.30	667.50
1-(3-methoxypropyl)-1-methylpyrrolidinium	tris(pentafluoroethyl)trifluorophosphate	1.47	5.15	4.31	23.30	603.30
1-(3-methoxypropyl)-1-methylpyrrolidinium	diperfluorophenyl-(triafluoro-3,5-dichlorophenyl)-methane	1.46	5.11	4.28	23.26	704.40
1-(3-methoxypropyl)-1-methylpyrrolidinium	triperfluorophenyl-methane	1.39	4.50	3.97	23.22	671.50
1-(3-methoxypropyl)-1-methylpyrrolidinium	diperfluorophenyl-(2,3,5,6-tetrafluorophenyl)-methane	1.41	4.67	4.05	23.19	653.50
4-(3-methoxypropyl)-4-methylmorpholinium	diperfluorophenyl-(2,3,5,6-tetrafluorophenyl)-methane	1.47	4.85	4.04	22.55	669.50
4-(3-methoxypropyl)-4-methylmorpholinium	diperfluorophenyl-(4-methyl-tetrafluorophenyl)-methane	1.48	4.87	4.05	22.52	683.50
4-(3-methoxypropyl)-4-methylmorpholinium	diperfluorophenyl-(triafluoro-3,5-dichlorophenyl)-methane	1.52	5.19	4.19	22.47	720.40
4-(3-methoxypropyl)-4-methylmorpholinium	triperfluorophenyl-methane	1.44	4.56	3.89	22.46	687.50
4-(3-methoxypropyl)-4-methylmorpholinium	diperfluorophenyl-(tetrafluoro-4-chlorophenyl)-methane	1.48	4.88	4.03	22.38	703.90
1-[2-[2-(2-methoxyethoxy)ethoxy]ethyl]pyridinium	tris(pentafluoroethyl)trifluorophosphate	1.33	4.10	3.78	23.22	671.30
1-[2-[2-(2-methoxyethoxy)ethoxy]ethyl]pyridinium	diperfluorophenyl-(4-methyl-tetrafluorophenyl)-methane	1.31	3.89	3.63	22.88	735.50
1-[2-[2-(2-methoxyethoxy)ethoxy]ethyl]pyridinium	diperfluorophenyl-(triafluoro-3,5-dichlorophenyl)-methane	1.35	4.14	3.75	22.87	772.40
1-[2-[2-(2-methoxyethoxy)ethoxy]ethyl]pyridinium	diperfluorophenyl-perchlorophenyl-methane	1.47	4.93	4.13	22.83	821.80
1-[2-[2-(2-methoxyethoxy)ethoxy]ethyl]pyridinium	diperfluorophenyl-(2,3,5,6-tetrafluorophenyl)-methane	1.31	3.85	3.61	22.83	721.50
ethyl-(3-methoxypropyl)-dimethylammonium	tris(pentafluoroethyl)trifluorophosphate	1.48	4.94	4.09	22.57	591.30
ethyl-(3-methoxypropyl)-dimethylammonium	diperfluorophenyl-(4-methyl-tetrafluorophenyl)-methane	1.42	4.46	3.86	22.55	655.50
ethyl-(3-methoxypropyl)-dimethylammonium	diperfluorophenyl-(triafluoro-3,5-dichlorophenyl)-methane	1.46	4.77	4.00	22.55	692.40
ethyl-(3-methoxypropyl)-dimethylammonium	diperfluorophenyl-(2,3,5,6-tetrafluorophenyl)-methane	1.41	4.42	3.83	22.50	641.50
ethyl-(3-methoxypropyl)-dimethylammonium	triperfluorophenyl-methane	1.39	4.21	3.72	22.42	659.50
di[n-di(2-methoxyethyl)]]-n-dimethyl-guanidinium	sbf6	1.38	5.78	5.15	26.21	556.20
di[n-di(2-methoxyethyl)]]-n-dimethyl-guanidinium	tris(pentafluoroethyl)trifluorophosphate	1.25	4.53	4.43	26.10	765.40
di[n-di(2-methoxyethyl)]]-n-dimethyl-guanidinium	pf6	1.36	5.42	4.87	25.78	465.40
di[n-di(2-methoxyethyl)]]-n-dimethyl-guanidinium	asf6	1.44	6.19	5.28	25.69	509.30
di[n-di(2-methoxyethyl)]]-n-dimethyl-guanidinium	diperfluorophenyl-(4-methyl-tetrafluorophenyl)-methane	1.30	4.45	4.19	24.84	829.60

## 4 Conclusions

*R*-410A is a hydrofluorocarbon mixture currently being phased out due to the high global warming potential of its constituents, particularly *R*-125. To mitigate environmental harm, it is essential to reclaim *R*-410A and separate *R*-125 to prevent atmospheric release. However, the azeotropic nature of these mixtures poses significant challenges, making their separation both difficult and energy-intensive, and necessitating the careful selection of effective solvents. In this work, we employ data-driven modeling and analysis to identify clear structure–selectivity trends for *R*-32 and *R*-125 across a wide range of ionic liquids, thereby revealing promising candidates for energy-efficient separation of *R*-410A. This study represents a comprehensive computational screening of ionic liquids for this application. We analyzed a data set of 341,687 ionic liquids and salts. We applied dimensionality reduction techniques to systematically explore the ionic liquid chemical space. We computed room-temperature solubilities of *R*-32 and *R*-125 in all ionic liquid candidates. Furthermore, we developed machine learning models for fast solubility and selectivity prediction. We have made these models available for the community at https://github.com/aiftakher/HFC-IL-ActivityCoefficient. Our analysis found several insights. We observed that ILs with polar, hydrogen-bonding characteristics (often via their anions) exhibit higher *R*-32 solubility, whereas ILs that are nonpolar favor *R*-125. Using PCA and t-SNE, we visualized cluster of ILs in major cationic family. The developed machine learning models could efficiently predict the infinite dilution activity coefficients (one of the major properties that dictate solubility and phase equilibria) of *R*-32 and *R*-125 in ILs. We utilized the sigma profiles (often regarded as the universal molecular descriptor^38^) as input features, which allow for the rapid and generalizable prediction of infinite dilution activity coefficients of *R*-32 and *R*-125 in any ionic liquid. These models can further be used for screening ionic liquids to separate other multicomponent HFC constituents involving *R*-32 and *R*-125. We also proposed a new screening metric and identified a set of new promising ionic liquids for *R*-410A separation. The absolute differences in solubility, or the solubility gap overcomes the issues often encountered with traditional selectivity-based solvent selection. We also observed that the existing ionic liquids that are currently considered for *R*-410A separation reside in a concentrated narrow region of the broader design space of possible ionic liquids, and there is a significant opportunity to discover new ionic liquids with more desirable properties. While the current focus is on the prediction of infinite dilution activity coefficients, future work will include the extension of our framework to predict phase-equilibrium data for multicomponent refrigerant mixture, incorporating temperature and pressure dependencies. One can also integrate process-level technoeconomic and lifecycle assessments for a more comprehensive screening strategy. Finally, embedding physical constraints directly into the neural network architecture (e.g., via projection layers^41^ or PINNs) offers a promising avenue to further improve model interpretability and guarantee feasibility.

## Supplementary Material



## Data Availability

All scripts, data sets, and source code are available at https://github.com/aiftakher/HFC-IL-ActivityCoefficient.

## Notes

Henry’s constant and selectivity distribution across IL families, navigation of IL families and *R*-125 selective ILs using PCA and t-SNE, cation and anion distribution of ILs with greatest selectivities, and detailed statistical information regarding model training, robustness, and generalizability (PDF)
