# Supplementary material for: Design Space Exploration and Machine Learning Prediction of Hydrofluorocarbon Solubility in Ionic Liquids for Refrigerant Separation
Source: J Chem Inf Model. 2025 Aug 25;65(22):12168–78. doi: 10.1021/acs.jcim.5c01216 (PMC12648660; doi:10.1021/acs.jcim.5c01216)
Supplement: Supplementary file 1 [file ci5c01216_si_001.pdf]

Design Space Exploration and Machine Learning Prediction of Hydrofluorocarbon Solubility in Ionic Liquids for Refrigerant Separation

Ashfaq Iftakher[1] and M. M. Faruque Hasan[1,2,*]

[1]Artie McFerrin Department of Chemical Engineering, Texas A&M University, College Station, TX 77843-3122, USA.

[2]Texas A&M Energy Institute, Texas A&M University, College Station, TX, 77843, USA.

KEYWORDS. HFC Separation, Machine Learning, Activity Coefficient, Refrigerants, Ionic Liquids

Supporting Information

S1. Experimental Validation of COSMO-RS Predictions

Table S1: List of Experimental and COSMO-calculated Henry's constants.

IL Name	T (K)	Source	H_{R32}^{exp} (MPa)	H_{R32}^{cal} (MPa)	H_{R125}^{exp} (MPa)	H_{R125}^{cal} (MPa)
1-ethyl-3-methylimidazolium Thiocyanate	283.15	(Asensio-Delgado et al., 2021a, 2021b)	2.61	2.33	12.9	7.71
1-ethyl-3-methylimidazolium Thiocyanate	293.15	(Asensio-Delgado et al., 2021a, 2021b)	3.16	2.94	17.6	9.41
1-ethyl-3-methylimidazolium Thiocyanate	303.15	(Asensio-Delgado et al., 2021a, 2021b)	4.08	3.65	20.6	11.26
1-ethyl-3-methylimidazolium Thiocyanate	313.15	(Asensio-Delgado et al., 2021a, 2021b)	4.86	4.44	28.4	13.24
1-butyl-3-methylimidazolium Tetrafluoroborate	298.15	(Morais et al., 2020)	1.54	2.58	4.19	5.88
1-butyl-3-methylimidazolium Hexafluorophosphate	298.15	(Morais et al., 2020)	1.24	2.79	4.34	7.37
1-ethyl-3-methylimidazolium Trifluoromethansulfonate	303.15	(Sosa et al., 2019)	1.61	3.26	3.35	7.42
1-hexyl-3-methylimidazolium Trifluoromethansulfonate	303.15	(He et al., 2017)	1.59	2.66	2.43	4.03
1-hexyl-3-methylimidazolium Chloride	298.15	(Baca et al., 2021)	2	2.24	1.16	1.89
1-hexyl-3-methylimidazolium Bromine	298.15	(Baca et al., 2021)	1.71	2.38	2.56	2.46
1-ethyl-3-methylimidazolium Bis(trifluoromethylsulfonyl)imide	298.15	(Shiflett et al., 2006; Shiflett and Yokozeki, 2008)	1.09	2.47	1.76	4.76

1-ethyl-3-methylimidazolium Acetate	303.15	(Sosa et al., 2019)	1.53	3.14	1.85	3.79
1-ethyl-3-methylimidazolium Dicyanamide	283.15	(Asensio-Delgado et al., 2021b)	1.81	2.20	8.27	6.17
1-ethyl-3-methylimidazolium Dicyanamide	293.15	(Asensio-Delgado et al., 2021b)	2.41	2.76	11.14	7.47
1-ethyl-3-methylimidazolium Dicyanamide	303.15	(Asensio-Delgado et al., 2021b)	2.93	3.40	14.55	8.89
1-ethyl-3-methylimidazolium Dicyanamide	313.15	(Asensio-Delgado et al., 2021b)	3.66	4.13	18.86	10.41
1-ethyl-3-methylimidazolium Dicyanamide	323.15	(Asensio-Delgado et al., 2021b)	5.03	4.94	21.85	12.02
1-butyl-3-methylimidazolium Acetate	298.15	(Morais et al., 2020)	1.2	2.81	0.81	2.93
1-butyl-3-methylimidazolium Dicyanamide	283.15	(Asensio-Delgado et al., 2021b)	1.51	1.96	4.83	4.19
1-butyl-3-methylimidazolium Dicyanamide	293.15	(Asensio-Delgado et al., 2021b)	1.92	2.46	6.59	5.09
1-butyl-3-methylimidazolium Dicyanamide	303.15	(Asensio-Delgado et al., 2021b)	2.4	3.02	9.12	6.08
1-butyl-3-methylimidazolium Dicyanamide	313.15	(Asensio-Delgado et al., 2021b)	3.1	3.66	12.29	7.15
1-butyl-3-methylimidazolium Dicyanamide	323.15	(Asensio-Delgado et al., 2021b)	3.94	4.38	15.96	8.30
1-butyl-3-methylimidazolium Thiocyanate	298.15	(Asensio-Delgado et al., 2021b)	3.11	2.89	13.32	6.95
1-hexyl-3-methylimidazolium Tetrafluoroborate	303.15	(He et al., 2017)	1.44	2.60	3.19	4.71
1-hexyl-3-methylimidazolium Bis(trifluoromethylsulfonyl)imide	298.15	(He et al., 2017)	0.96	2.15	1.46	3.01
1-ethyl-3-methylimidazolium Perfluoropentanoate	303.15	(Sosa et al., 2019)	1.17	2.29	0.93	2.80
1-ethyl-3-methylimidazolium Perfluorobutanesulfonate	303.15	(Sosa et al., 2019)	1.15	2.31	1.29	2.97
Trihexyltetradecylphosphonium Chloride	298.15	(Baca et al., 2021)	0.63	2.02	0.37	1.12

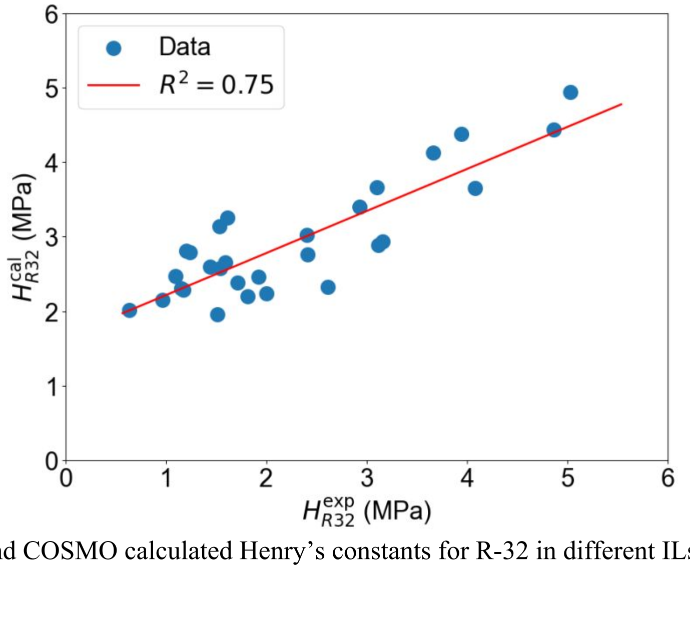

Figure S1: Experimental and COSMO calculated Henry's constants for R-32 in different ILs at different temperatures.

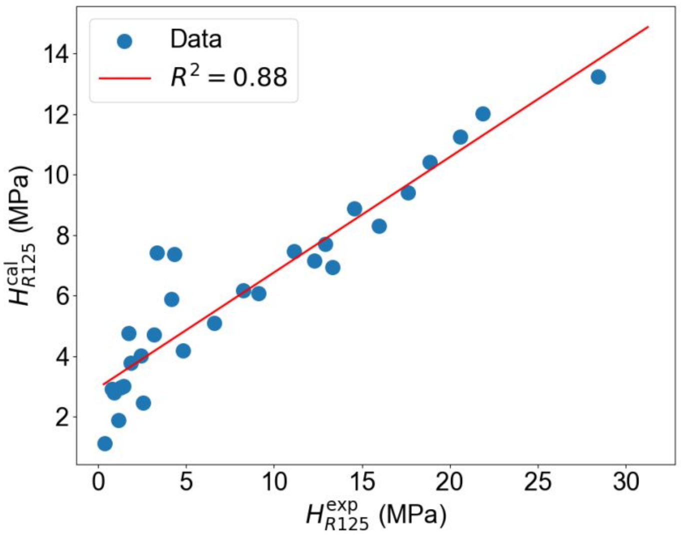

Figure S2: Experimental and COSMO calculated Henry's constants for R-125 in different ILs at different temperatures.

S2. Distribution of Ions, Henry's Constants and Selectivity in IL Families

Table S2. Number of ILs in major cationic families and ranges of R-32 selectivity.

Cation family	Number of ILs	Number of Cations	Maximum S_{R32}
Imidazolium	71161	143	37.05
Piperidinium	5512	11	12.76
Pyrrolidinium	9554	19	14.54
Morpholinium	4515	9	24.76
Pyridinium	33092	67	19.01
Ammonium	26978	54	32.91
Miscellaneous	190875	380	551.84

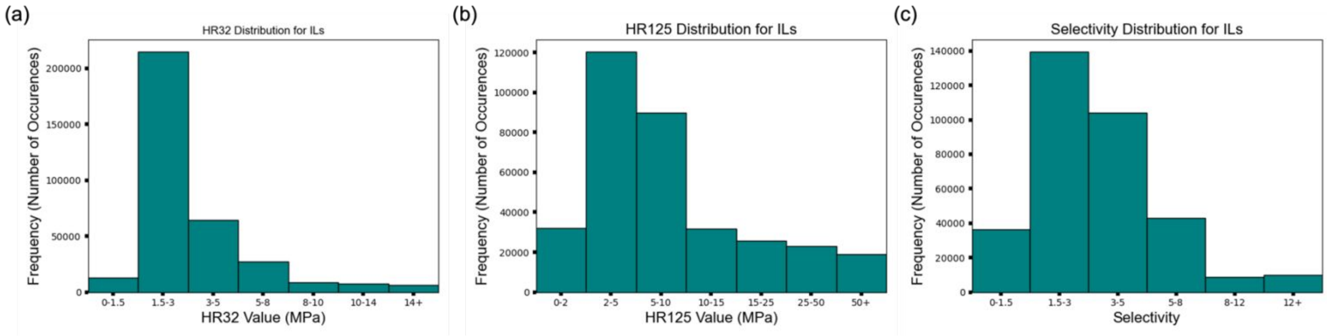

Figure S3: Henry's constants and R-32 selectivity distribution for all ILs.

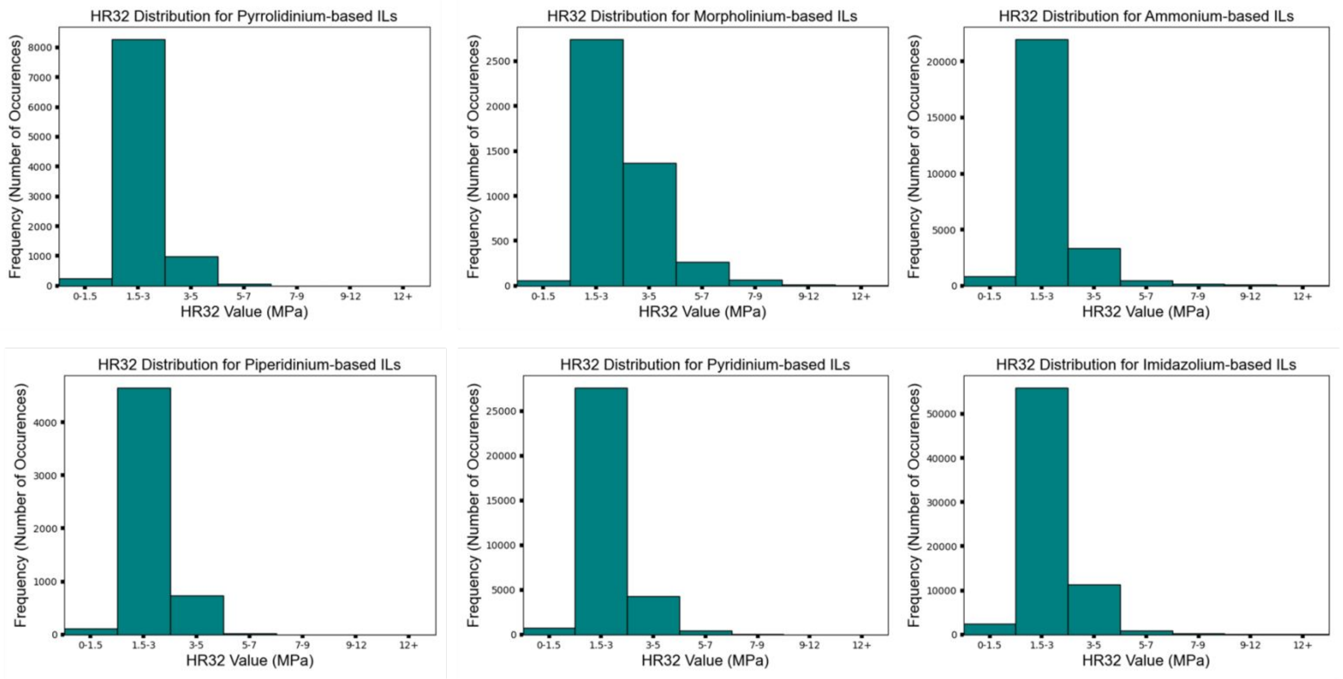

Figure S4: Henry’s constant of R-32 distribution for ILs in each major cationic family.

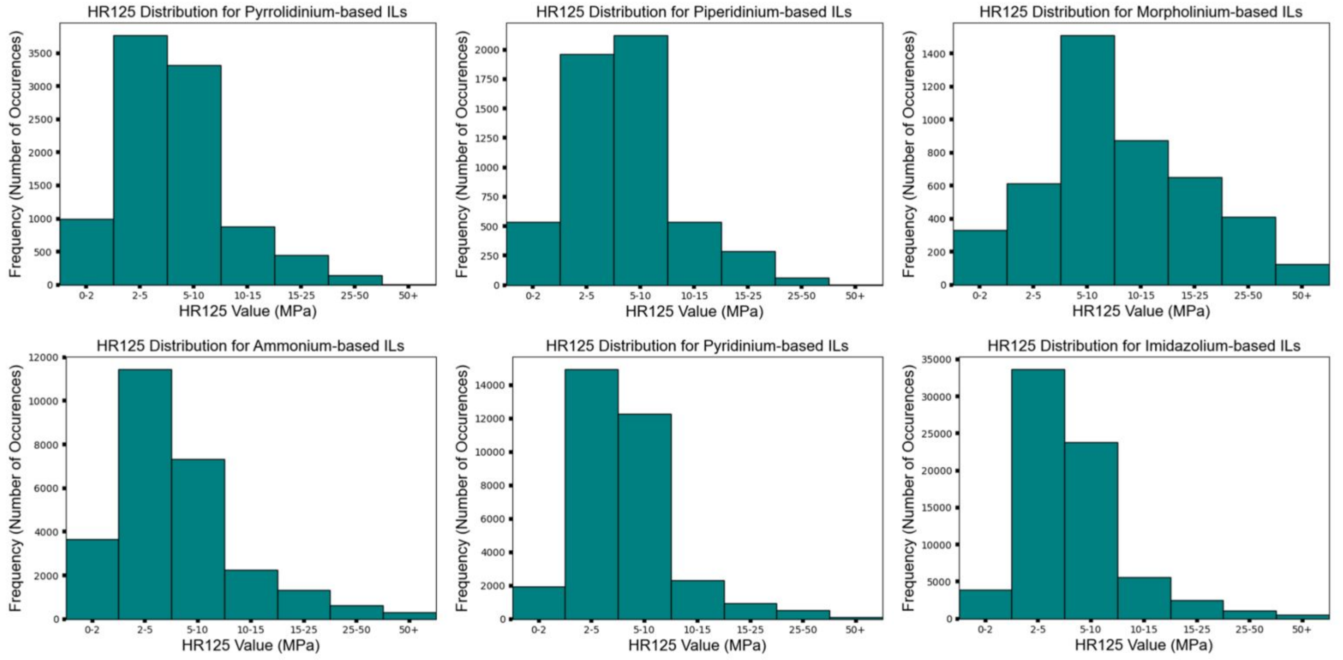

Figure S5: Henry’s constant of R-32 distribution for ILs in each major cationic family.

S3. Visualization of the IL chemical Space

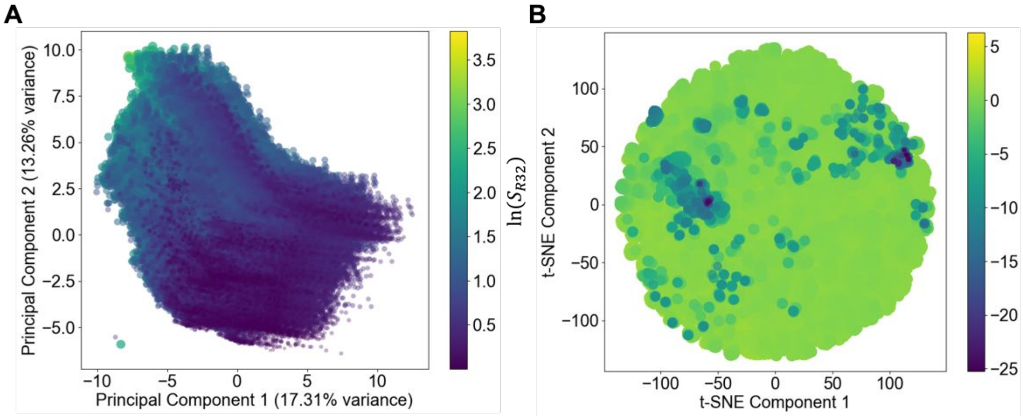

Figure S6: A two-dimensional projection of the chemical space using sigma profiles-based embeddings. (A) PCA of R-32 selective ILs with $H_{R32} \leq 10$ MPa and $S_{R32} > 1$, (B) t-SNE of the chemical space with colors based on the natural log of R-32 selectivity.

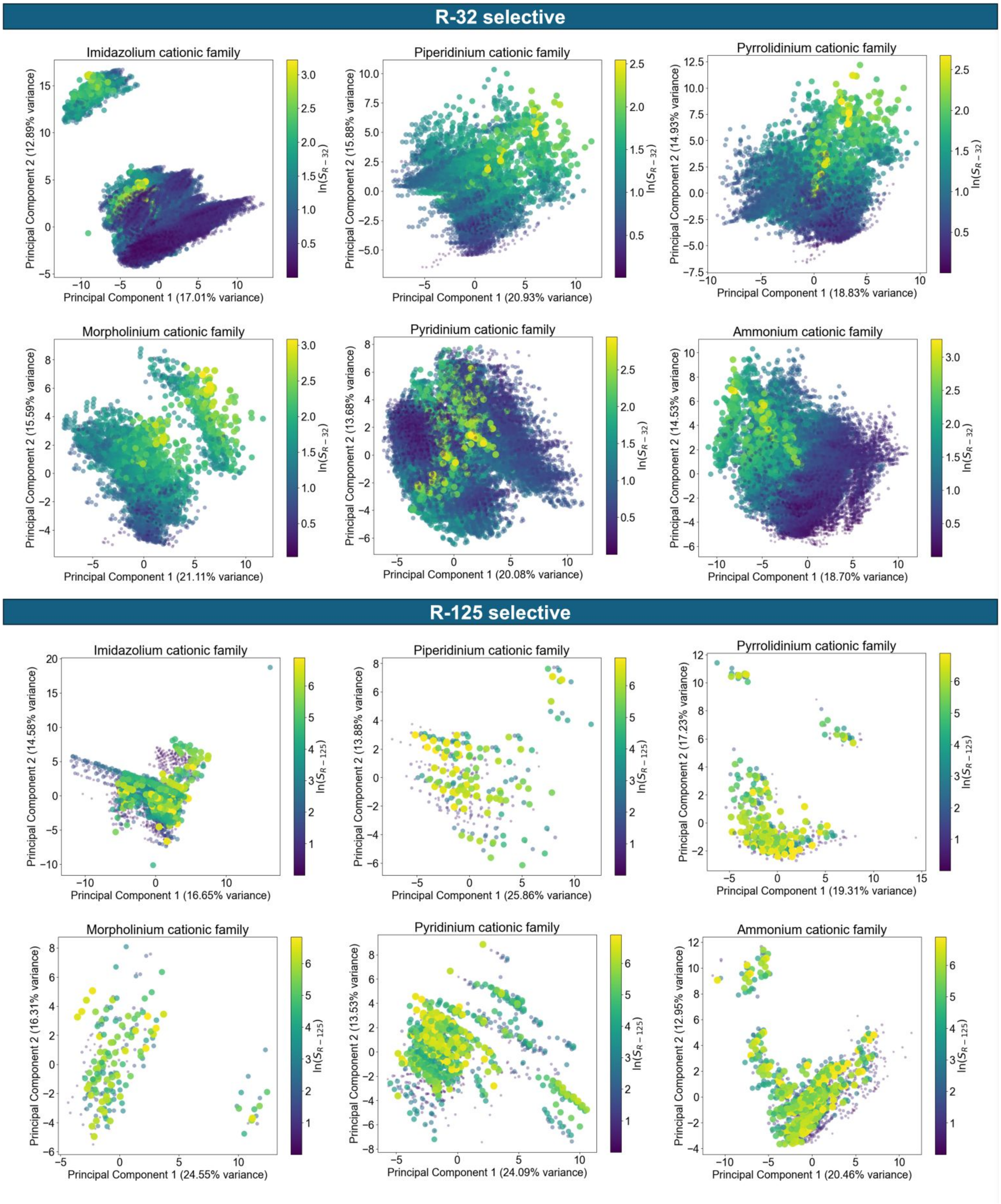

Figure S7. A two-dimensional projection of sigma profiles-based embeddings of IL chemical space using PCA. Each class of Cationic family is colored based on R-32 or R-125 selectivity.

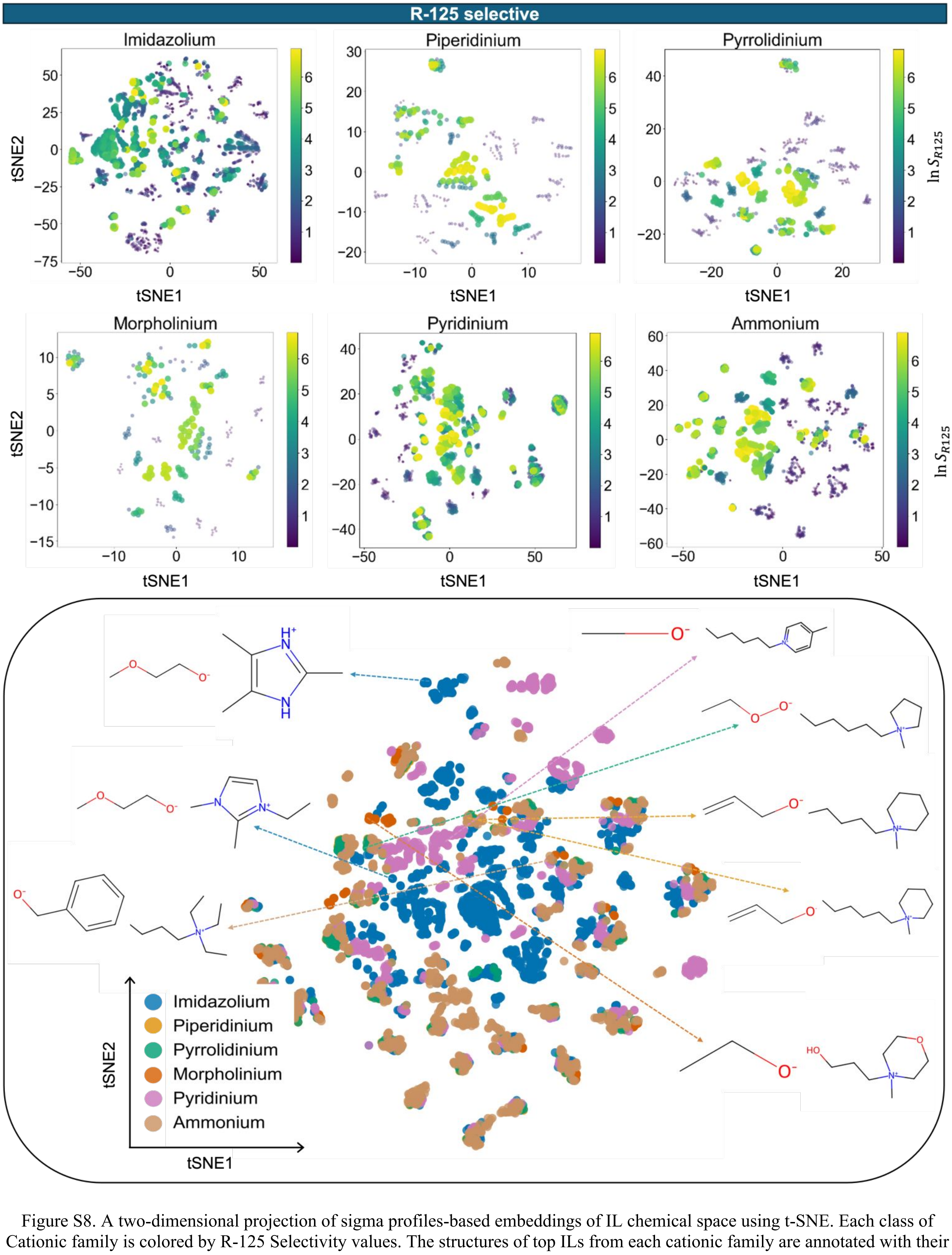

Figure S8. A two-dimensional projection of sigma profiles-based embeddings of IL chemical space using t-SNE. Each class of Cationic family is colored by R-125 Selectivity values. The structures of top ILs from each cationic family are annotated with their structures in the embedding space. For t-SNE visualization of R-125 selective ILs, top 5000 R-125 selective ILs are chosen with $H_{R125} \leq 10$ MPa and $1 \leq S_{R125} \leq 1000$.

S4. Cations and Anions of selected ILs based on R-32 selectivity

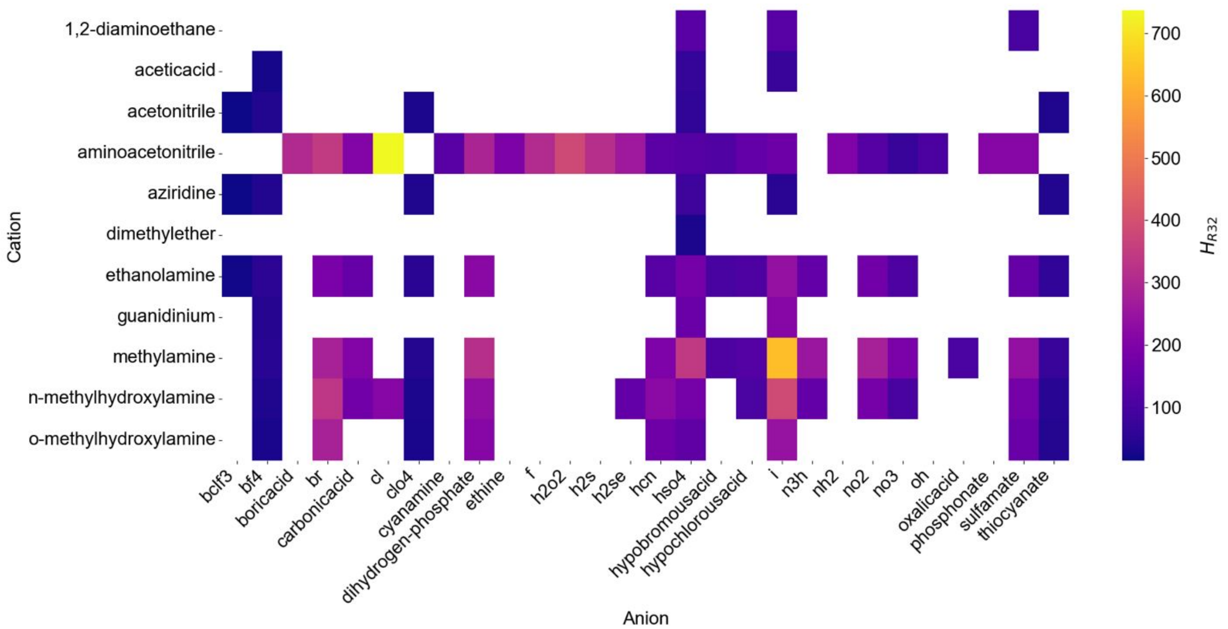

Figure S9. Heatmap of the Henry's constant of top 100 ILs based on R-32 selectivity.

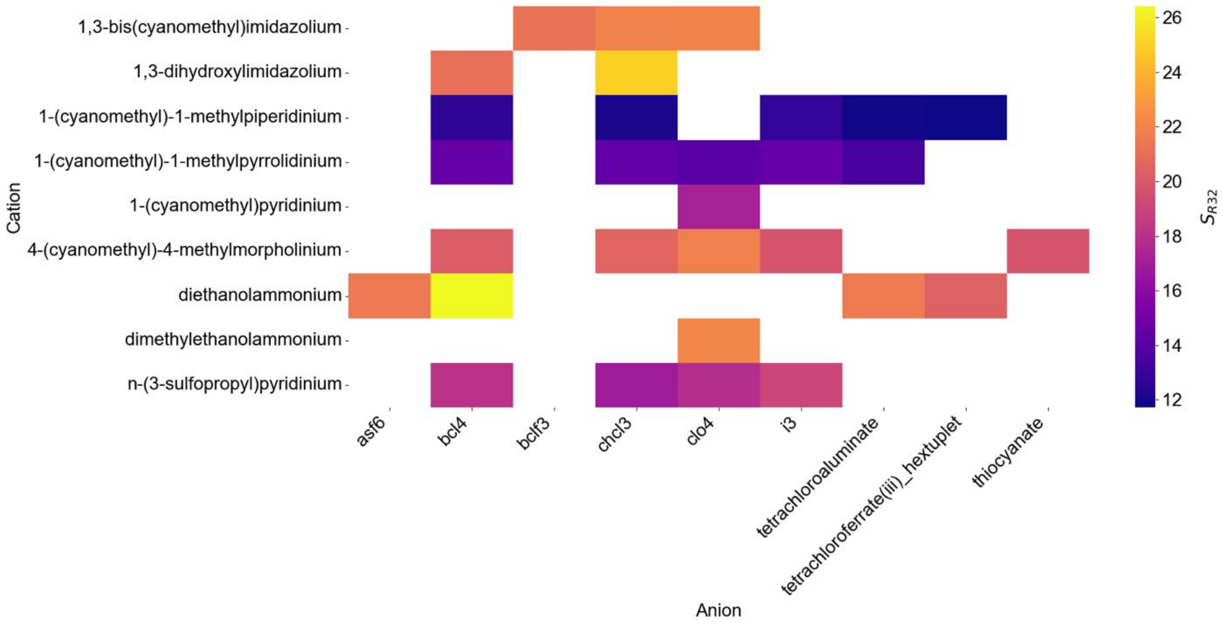

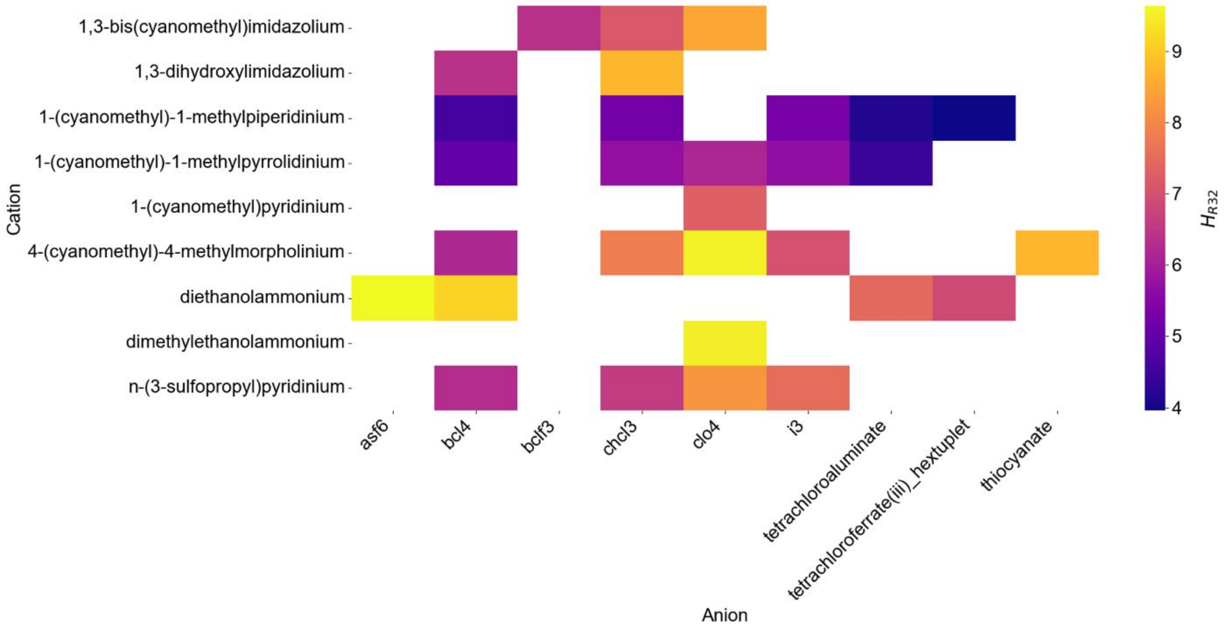

Figure S10. Heatmap of top 5 ILs from each cationic family based on R-32 selectivity.

S5. Additional Model Statistics

ANN Model for R-32 Selectivity:

5-Fold CV Mean R^2 = 0.984 ± 0.001

5-Fold CV Mean RMSE = 0.204 ± 0.009

Table S3: 5-fold cross validation results for the ANN model for R-32 selectivity.

Fold	R^2	RMSE
1	0.982	0.217
2	0.984	0.208
3	0.983	0.208
4	0.986	0.194
5	0.986	0.194

Statistical Indicators of the log Selectivity Model:

Training set:

R^2 : 0.990

RMSE : 0.160

MAE : 0.087

MAPE : 17.8%

RRMSE : 18.6%

Test Set
R^2 : 0.983
RMSE : 0.210
MAE : 0.094
MAPE : 45.9%
RRMSE : 24.2%

Statistical Indicators of the linear model for selectivity:
We have also trained and evaluated an identical ANN on raw S (no log transform). On the test set, it achieves:

R^2 = 0.756
RMSE = 2.269
RRMSE = 61.9 %

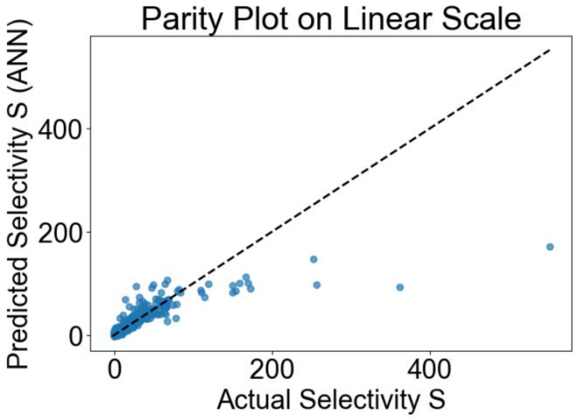

Figure S11: Parity plot for the linear scale of selectivity

Special Case of Leaving out ILs with PF_6 anions:

Number of test ILs: 681,
R^2 = 0.826
RMSE = 0.255
MAE = 0.240
MAPE = 15.9%
RRMSE = 3.9%

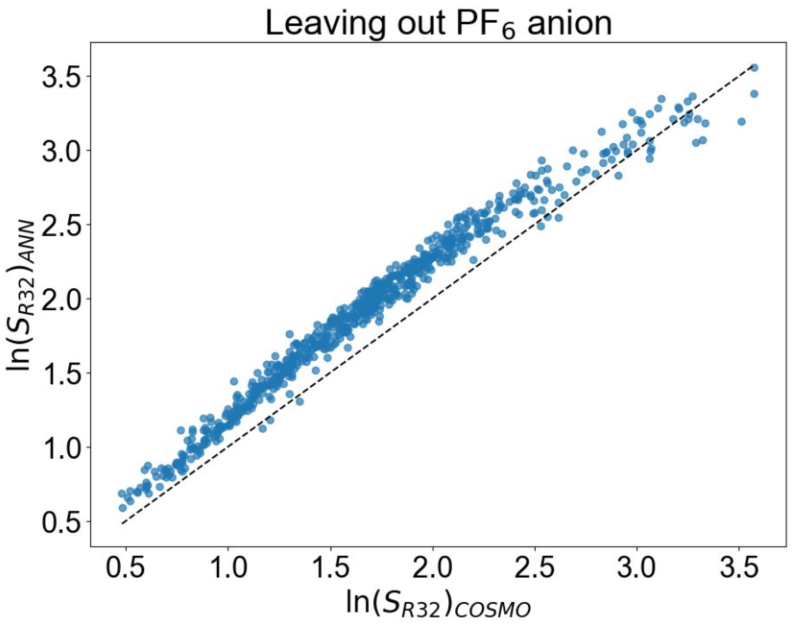

Figure S12: Parity plot for the selectivity prediction for PF_6 anion on the logarithmic scale

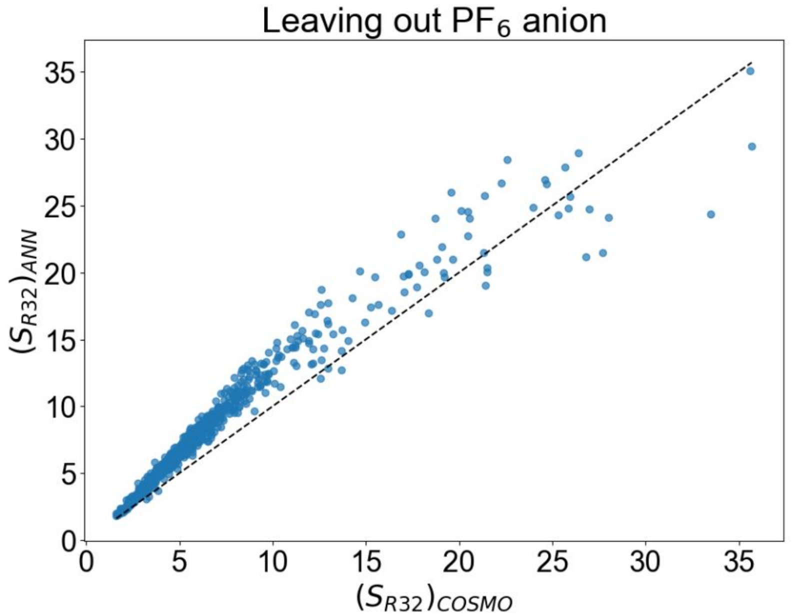

Figure S13: Parity plot for the selectivity prediction for PF_6 anion on the linear scale

Data Split Information:

Table S4: Data set summary for regression models.

Model	Split	No. of samples	Mean	Standard Deviation
lnGamma_R32	Train	218679	0.5218	0.5717
lnGamma_R32	Validation	54670	0.5184	0.5687
lnGamma_R32	Test	68338	0.5240	0.5690
lnGamma_R125	Train	218679	1.3827	1.8719
lnGamma_R125	Validation	54670	1.3813	1.8695
lnGamma_R125	Test	68338	1.3911	1.8514
lnSelectivity	Train	218679	0.8610	1.6401
lnSelectivity	Validation	54670	0.8629	1.6369
lnSelectivity	Test	68338	0.8671	1.6150

Binary Classifier Model Information:

Table S5: Data set summary for classification models.

Model	Split	No. of samples	No. of R32 selective ILs (%R32)	No of R125 selective ILs (%R125)
Classification	Train	218679	205636 (94.0%)	13043 (6.0%)
Classification	Validation	54670	51440 (94.1%)	3230 (5.9%)
Classification	Test	68338	64276 (94.1%)	4062 (5.9%)

Baseline model performance on the test set:
Accuracy = 0.995; Precision = 0.997; Recall = 0.998; AUC = 0.999.
Regularized model performance (Dropout = 0.2, L2 = 1×10^{-4}):
Accuracy = 0.992; Precision = 0.994; Recall = 0.997; AUC = 0.999),

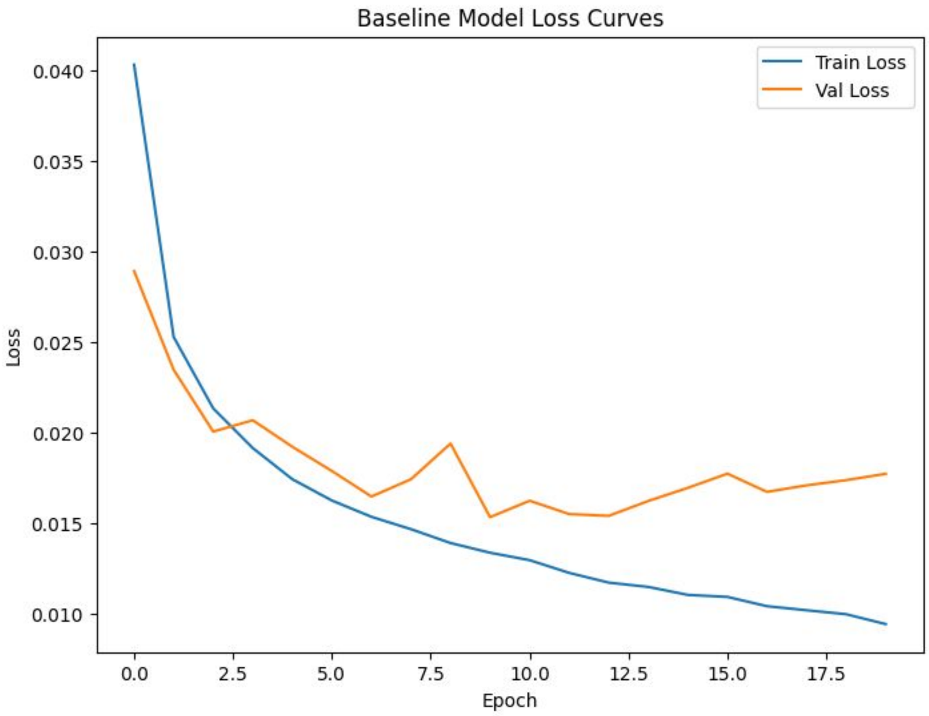

Figure S14. Loss curve of the binary classifier model.

Table S6: Five-fold cross-validation metrics for the classification model

5-Fold CV	Averages
Accuracy	0.995 +- 0.000
Precision	0.997 +- 0.000
Recall	0.998 +- 0.000

SHAP analysis of the R-32 selectivity ANN:

Directionality. The beeswarm in Fig. S15 shows how high (pink) vs. low (blue) values of the top 20 bins influence the prediction of $\ln S_{R32}$, moving it up or down.

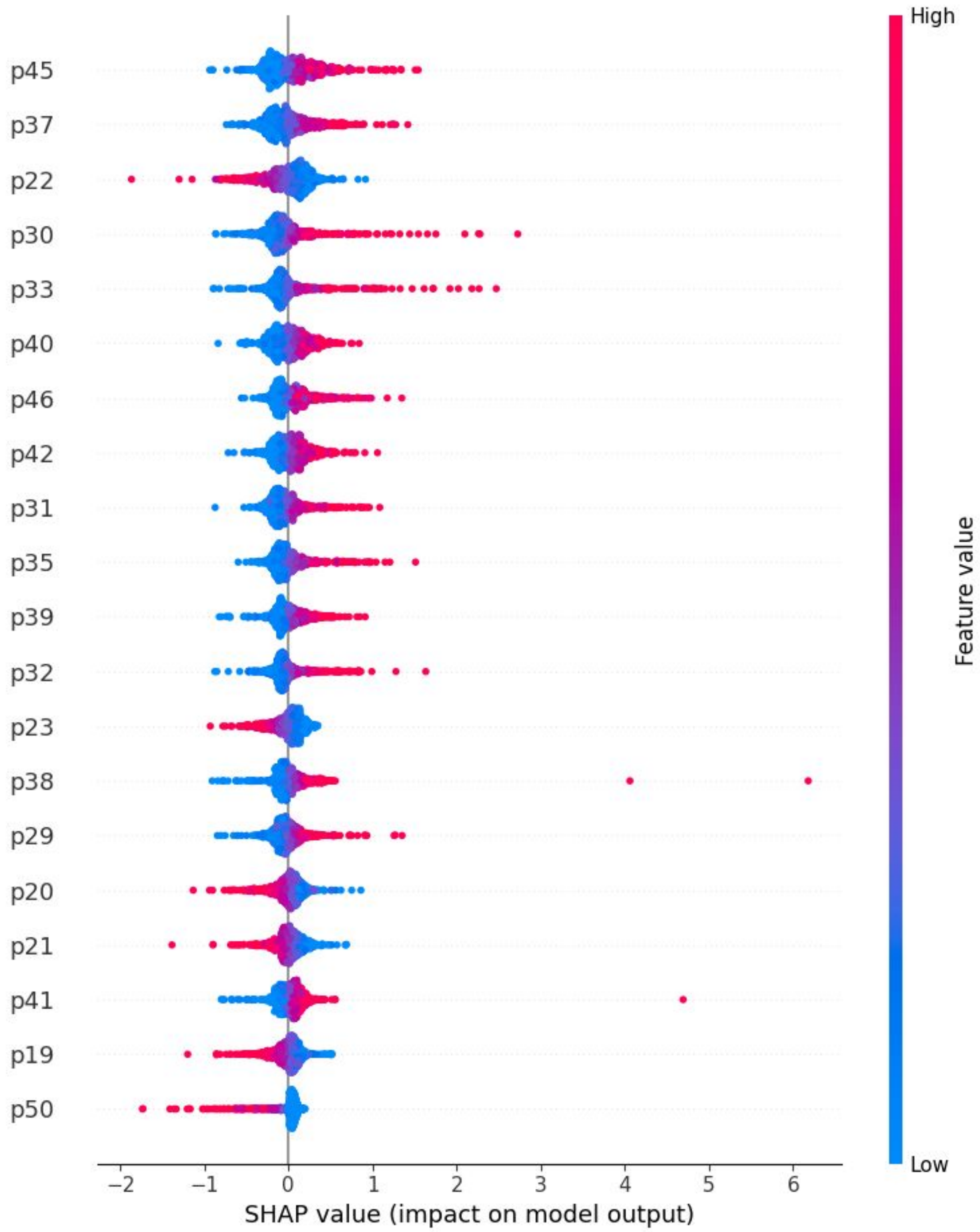

Figure S15. SHAP beeswarm for the R-32 selectivity ANN (evaluation n=1,000). Points show feature-level contributions and color encodes the original (standardized) bin value.

Global importance.

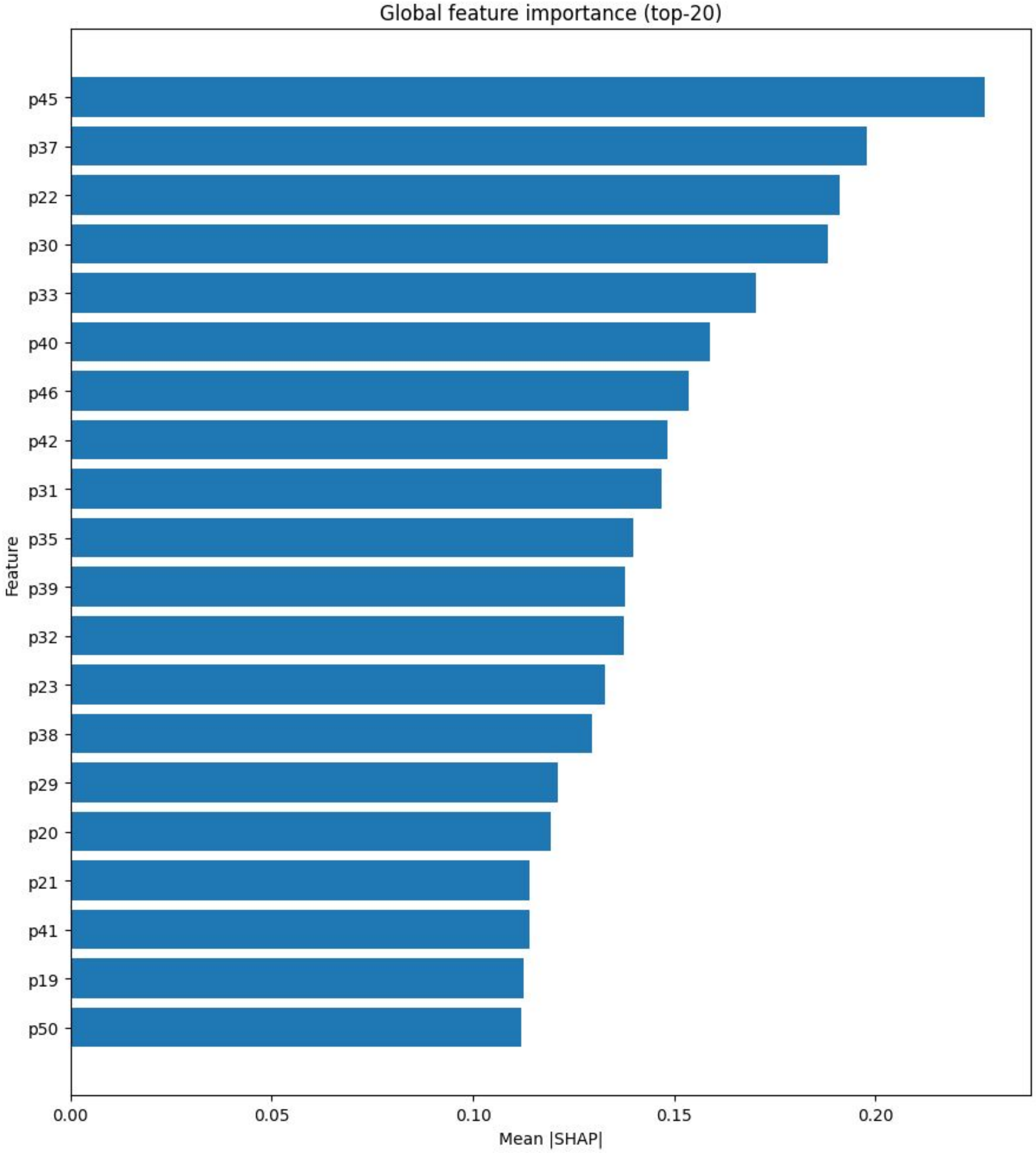

Figure S16: Global feature importance (mean absolute SHAP value per σ-bin) of the top 20 bins.

Top-5 share = 22.7%
Top-10 share = 40.2%
Top-20 share = 68.9%
k80 (features to reach 80%): 25
Effective features (entropy): 38.4

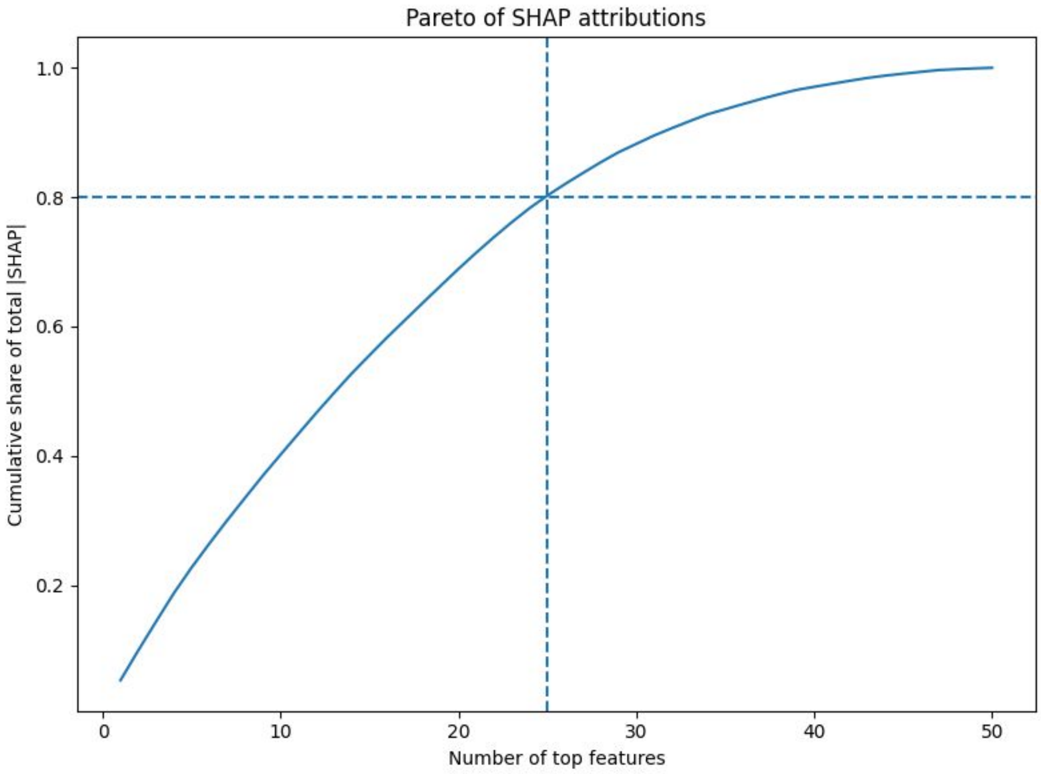

Figure S17: Pareto of SHAP attributions with 80% line and k80 = 25 marked.

The top 10 bins by mean SHAP are p45, p37, p22, p30, p33, p40, p46, p42, p31, p35. The values and type of the polar region are given in Table S7.

Table S7: Top 10 features, type and the mean SHAP

Feature	Type of polar region	Mean $\|SHAP\|$
P45	Positive	0.227
P37	Positive	0.198
P22	Nonpolar	0.191
P30	Nonpolar	0.188
P33	Nonpolar	0.171
P40	Positive	0.159
P46	Positive	0.154
P42	Positive	0.148
P31	Nonpolar	0.147
P35	Positive	0.139

Reference:

Asensio-Delgado, S., Pardo, F., Zarca, G., Urtiaga, A., 2021a. Absorption separation of fluorinated refrigerant gases with ionic liquids: Equilibrium, mass transport, and process design. Separation and Purification Technology 276, 119363. https://doi.org/10.1016/j.seppur.2021.119363

Asensio-Delgado, S., Viar, M., Pardo, F., Zarca, G., Urtiaga, A., 2021b. Gas solubility and diffusivity of hydrofluorocarbons and hydrofluoroolefins in cyanide-based ionic liquids for the separation of refrigerant mixtures. Fluid Phase Equilibria 549, 113210.

Baca, K.R., Olsen, G.M., Matamoros Valenciano, L., Bennett, M.G., Haggard, D.M., Befort, B.J., Garciadiego, A., Dowling, A.W., Maginn, E.J., Shiflett, M.B., 2021. Phase Equilibria and Diffusivities of HFC-32 and HFC-125 in Ionic Liquids for the Separation of R-410A. ACS Sustainable Chemistry & Engineering 10, 816–830.

He, M., Peng, S., Liu, X., Pan, P., He, Y., 2017. Diffusion coefficients and Henry's constants of hydrofluorocarbons in [HMIM][Tf2N],[HMIM][TfO], and [HMIM][BF4]. The Journal of Chemical Thermodynamics 112, 43–51.

Morais, A.R.C., Harders, A.N., Baca, K.R., Olsen, G.M., Befort, B.J., Dowling, A.W., Maginn, E.J., Shiflett, M.B., 2020. Phase Equilibria, Diffusivities, and Equation of State Modeling of HFC-32 and HFC-125 in Imidazolium-Based Ionic Liquids for the Separation of R-410A. Industrial & Engineering Chemistry Research 59, 18222–18235. https://doi.org/10.1021/acs.iecr.0c02820

Shiflett, M.B., Harmer, M.A., Junk, C.P., Yokozeki, A., 2006. Solubility and diffusivity of difluoromethane in room-temperature ionic liquids. Journal of Chemical & Engineering Data 51, 483–495.

Shiflett, M.B., Yokozeki, A., 2008. Binary vapor–liquid and vapor–liquid–liquid equilibria of hydrofluorocarbons (HFC-125 and HFC-143a) and hydrofluoroethers (HFE-125 and HFE-143a) with ionic liquid [emim][Tf_2N]. Journal of Chemical & Engineering Data 53, 492–497.

Sosa, J.E., Ribeiro, R.P., Castro, P.J., Mota, J.P., Araújo, J.M., Pereiro, A.B., 2019. Absorption of fluorinated greenhouse gases using fluorinated ionic liquids. Industrial & Engineering Chemistry Research 58, 20769–20778.
